# The Association Between Serum Gamma‐Glutamyl Transferase and Gastrointestinal Cancer Risk: A Systematic Review and Meta‐Analysis

**DOI:** 10.1002/cam4.70581

**Published:** 2025-01-16

**Authors:** Alireza Ramandi, Jacob George, Amir Hossein Behnoush, Alireza Delavari, Zahra Mohammadi, Hossein Poustchi, Reza Malekzadeh

**Affiliations:** ^1^ Division of Gastroenterology and Hepatology Weill Cornell Medicine New York New York USA; ^2^ Digestive Disease Research Center Digestive Disease Research Institute, Tehran University of Medical Sciences, Shariati Hospital Tehran Iran; ^3^ Storr Liver Centre Westmead Institute for Medical Research, Westmead Hospital and University of Sydney Westmead New South Wales Australia; ^4^ Liver and Pancreatobiliary Diseases Research Center, Digestive Diseases Research Institute Tehran University of Medical Sciences Tehran Iran

**Keywords:** cancer, gamma‐glutamyl transferase (GGT), gastrointestinal, incidence, meta‐analysis, systematic review

## Abstract

**Background:**

Gamma‐glutamyl transferase (GGT) has been shown to have associations with several diseases including cancers. Previous studies have investigated the effect of GGT levels on the gastrointestinal (GI) cancer incidence. We aim to systematically investigate these studies to provide better insights into the interrelationship between GGT and GI cancers.

**Methods:**

Online databases were searched to find relevant studies investigating different GGT levels' effects on the incidence of GI cancers including colorectal, esophageal, liver, pancreas, gastric, and biliary duct cancers. Random‐effect meta‐analysis was conducted to pool the hazard ratios (HRs) of GGT quartiles (Qs) effect on cancer incidence.

**Results:**

A total of 26 studies were included in the final review, 12 of which underwent meta‐analysis that investigated 11 million patients. Based on the meta‐analysis, Q4 patients had a 69% higher hazard of GI cancer incidence (HR 1.69, 95% CI 1.41–2.02, *p*‐value < 0.001). The hazard ratio significance was also similar for Q3 (HR 1.22, 95% CI 1.15–1.30, *p*‐value < 0.001) and Q2 (HR 1.10, 95% CI 1.05–1.16, *p*‐value =0.002) of GGT. Colorectal and liver cancers showed a higher hazard ratio among Q2, Q3, and Q4 of GGT compared to Q1. In pancreas and bile duct cancers, only Q4 of GGT had significantly higher HR. Q3 and Q4 of GGT levels had statistically significant associations with gastric cancer incidence.

**Conclusion:**

Higher GGT levels correlate with higher rates of GI cancer incidence, especially in colorectal and hepatic cancers. Future studies should investigate this biomarker's potential role in risk assessment for digestive cancers.

## Graphical abstract

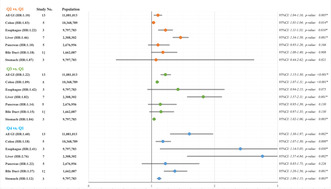

## 1 Introduction

Gamma‐glutamyl transferase (GGT) is a liver enzyme present in other tissues and a marker for several diseases [1]. Being abundant in digestive tract tissues, GGT has historically correlated with a handful of conditions such as alcohol intake [2, 3]. However, the recent literature has highlighted GGT as a systemic inflammatory marker for an array of chronic disorders, spanning from insulin resistance to cardiovascular diseases and cancer [1, 4, 5, 6, 7, 8, 9, 10]. Higher GGT levels are associated with a higher risk of breast, lung, prostate, and GI tract cancers [11, 12, 13, 14]. This association may stem from the protective role of the glutathione metabolism pathway or from an as‐yet‐unclarified cellular mechanism that involves cancer pathogenesis [8].

GGT has been tested as a biomarker for cancer diagnosis, prognosis, and prediction, especially in digestive system cancers [2, 3]. These cancers are either among the most prevalent (e.g., colon, stomach, and liver cancers) or among the most insidious (e.g., esophagus and pancreas) [15, 16, 17, 18, 19, 20, 21, 22, 23, 24, 25]. Thus, providing early clues of their incidence is of very high value.

GGT's association with cancer, however, remains inconsistent across different reports. It is also unclear whether GGT directly contributes to cancer pathogenesis or merely serves as an inflammatory marker elevated in response to homeostatic disruption. We aimed to calculate the pooled predictive value of GGT for digestive cancer incidence by systematically reviewing the current literature. Total GI cancer incidence and specific cancer site incidences were investigated. We also sought to clarify the cancer signaling pathways involving GGT by integrating current molecular findings into a comprehensive model.

## 2 Methods

This systematic review and meta‐analysis followed the Preferred Reporting Items for Systematic Reviews and Meta‐Analyses (PRISMA) 2020 guidelines [26]. The protocol was registered in PROSPERO (CRD42024551577).

### 2.1 Literature Search and Screening

We systematically searched PubMed, Embase, and Scopus databases for studies published before May 1, 2024. Our PECOT‐structured search strategy consisted of the general adult population (P) with GGT level assessment (E) who were followed for any new‐onset gastrointestinal cancer (O) according to the International Classification of Diseases 10th Revision (ICD‐10‐C15‐26). We aimed to find cohort studies in which different GGT levels served as the comparison groups (C) for the determination of cancer incidence. We performed multiple pilot searches and added newly found keywords to the search index until no further articles were found. Search terms included “Gamma‐glutamyl transferase” AND “gastrointestinal cancer” as well as their related terms. Supplementary Table 1 shows all the search terms and their relative retrieved numbers of studies.

### 2.2 Data Extraction

Following duplicate data removal, two independent authors (A.R. and A.H.B.) screened the title‐abstracts of the preliminary search results. Any uncertainties were retained for further full‐text assessment. The third author (J.G.) resolved any discrepancies between the two authors following full‐text evaluation. The reliability of title‐abstract screening was validated by two pilot screening tests, each comprising 10% of the total articles. Two authors independently evaluated the full texts that passed screening. Authors extracted data using a pre‐designed data sheet including (1) first author, year, country, and database name; (2) cancer site, follow‐up period, GGT quartiles (Q1–Q4), mean age, and sex proportions; and (3) hazard ratio (HR), hazard ratio standard error, and 95% confidence interval. Any missing values in reported HRs were calculated based on the remaining available data using Parmar et al.'s method [27]. Every step of the screening process is reported using Preferred Reporting Items for Systematic Reviews and Meta‐Analyses (PRISMA) guidelines [26]. Each excluded article is annotated with a reason, except those excluded during the title‐abstract screening phase.

### 2.3 Quality Appraisal

We used the Newcastle‐Ottawa Scale (NOS) to assess the quality of the included studies [28]. Briefly, this protocol scores studies based on selection bias, comparability level, and outcome quality. The studies are then categorized into good, fair, and low quality based on the cumulative score. The quality appraisal was conducted by author HP, who was not involved in data extraction.

### 2.4 Statistical Analysis

We used R version 4.3 (R Core Team [2020]. R: A language and environment for statistical computing. R Foundation for Statistical Computing, Vienna, Austria) for our statistical analysis. Meta‐analysis was coded with the ‘meta’, ‘metafor’, and ‘rmeta’ libraries. Meta‐analysis of hazard ratios (HRs) and their 95% confidence intervals (CIs) was performed by using the random‐effect method due to the variations in exposure and outcome assessment among different studies [29]. Tau statistics for random treatment effects were calculated by the DerSimonian‐Laird method [30]. To keep the analysis accurate and conservative, we utilized the conservative Hartung‐Knapp modification [31, 32]. Minor quartile variations were corrected before pooled analysis by using the meta‐analysis missing values algorithm [33].

The heterogeneity was evaluated by Cochran's *Q* statistics and Higgins' *I*‐square test *Q* [34, 35]. *I* ^2^ ranges of 0–25, 26–75, and 75–100 were considered as low, moderate, and high heterogeneity levels, respectively. We sought to report Tau statistics to avoid any bias caused by study size or count [30]. Funnel plot and Egger's test were used to report possible publication bias. We corrected our study results for publication bias by using trim‐and‐fill and Copas methods [33, 36, 37]. Briefly, trim‐and‐fill aims to reduce selection bias by trimming outlier studies followed by re‐adding the study and its mirror to the remainder of the studies. Copas modeling does not aim to reduce selection bias but seeks to adjust publication bias in the meta‐analysis.

### 2.5 Ethical Considerations

The study protocol for this review was performed in accordance with PROSPERO guidelines and reporting system and approved by the review board of the Digestive Diseases Research Institute and ethics committees at Tehran University of Medical Science (IR.TUMS.MEDICINE.REC.1401.701).

## 3 Results

### 3.1 Literature Search and Included Studies

**FIGURE 1:**
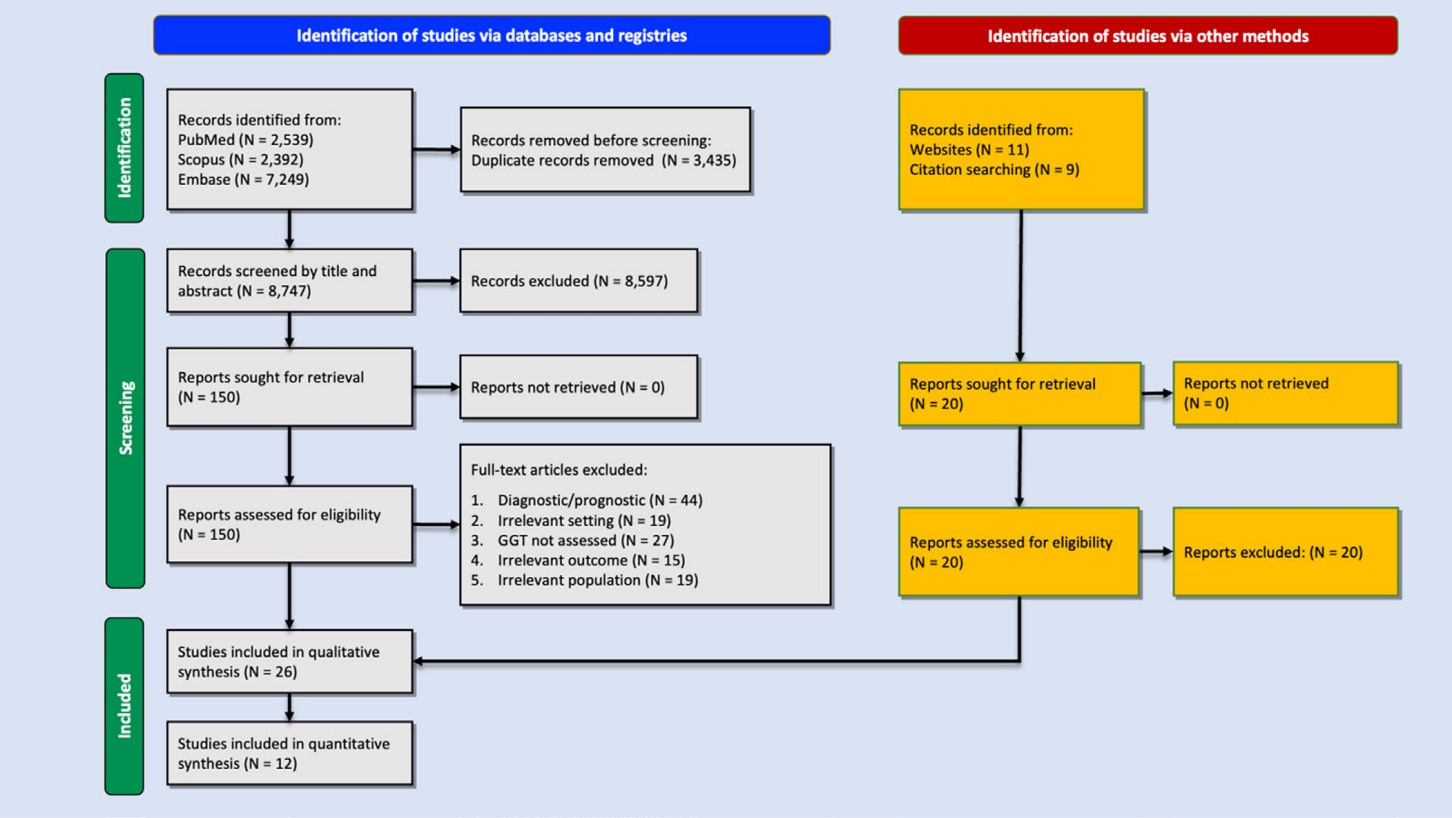
PRISMA flowchart showing the search and study selection process.

Our search strategy identified 12,182 articles, including 2539 from PubMed, 7249 from Embase, and 2392 from Scopus. After the removal of duplicates (*n* = 3435), 8747 studies underwent title/abstract screening, after which 150 studies remained. These were reviewed in full text. Finally, 26 articles were included for qualitative synthesis [11, 12, 13, 38, 39, 40, 41, 42, 43, 44, 45, 46, 47, 48, 49, 50, 51, 52, 53, 54, 55, 56, 57, 58, 59, 60], 12 of which were also considered for quantitative synthesis [11, 12, 13, 46, 48, 49, 50, 52, 56, 57, 58, 59]. Figure 1 illustrates the database search and study selection process using a PRISMA flowchart. The screen‐accepted article count was in line with the pilot search and screens (*p* = 0.907).

Newcastle‐Ottawa quality appraisal resulted in 17 high‐quality [11, 12, 13, 38, 40, 42, 43, 45, 46, 48, 49, 50, 51, 52, 57, 58, 59] and 9 medium‐quality studies [39, 41, 44, 47, 53, 54, 55, 60] (Figure S1). The most common violated concerns in the quality appraisal were the lack of sufficient covariate inclusion and insufficient representativeness of subjects. 11,080,650 patients from non‐overlapping study designs were included, spanning colorectal, gastric, esophageal, hepatic, pancreatic, and bile duct neoplasms.

### 3.2 Digestive Tract Cancers

Our qualitative synthesis extracted 11 articles that addressed all digestive cancers as the primary outcome of their study, including seven and four studies on cancer incidence [11, 12, 13, 46, 51, 58, 59] and cancer mortality [40, 41, 42, 53], respectively. Two cancer mortality studies modeled the dose–response association of GGT levels in survival analysis, one of which reported an HR of 1.002 (95% CI 1.001–1.003) per one unit increase of GGT over 15 years of follow‐up [41], while the other reported an HR of 1.4 (95% CI 1.02–1.94) over 21.5 years of follow‐up [53] (Table 1). Both studies concluded that GGT level is associated with cancer‐related mortality in a dose–response manner. The rest of the cancer mortality studies were in line with the mentioned findings. The current literature lacked studies addressing cancer incidence as a dose–response association with baseline GGT. Nevertheless, we found seven studies that addressed GGT levels as quartiles, all of which implied a significant increase in cancer incidence in Q4 of GGT levels. Subjects with Q2 and Q3 GGT levels showed significant increases in cancer incidence in two [12, 13] and three studies [12, 13, 46], respectively.

**TABLE 1:** Characteristics of included studies in the systematic review.

Author (year)	Country (database)	Participants	Comparison group (GGT measurement)	Follow‐up (years)	Outcome
Ahmed, M (2023) [38]	United Kingdom (Biobank)	Prospective cohort with 290,888—participants aged 37–73	Patients with high GGT and LFT, BS, and TG as a group compared to patients with normal metabolic biomarkers, low BMI, high HDL. GGT analyzed as a continuous variable	6–10	Hazard ratio of hepatocellular liver cancer was higher in exposure arm of the study, compared to the control group (GGT HR = 2.40; 95% CI = 2.19–2.65) Cholangiocarcinoma incidence was also higher in High GGT+ High LFT+ group compared to the control (GGT HR = 3.27; 95% CI = 1.47–2.11)
Bai, C (2022) [39]	China (Qingdao)	Nested Case–control study with 168,858 cancer patients and 132,357 healthy controls	Subjects with Lowest and highest 2.5% GGT were excluded. GGT analyzed as quartiles; First quartile used as the reference group for the rest of the quartiles	5	Higher GGT levels were observed in Patients with colon cancer (*p*‐value = 0.001), rectum cancer (*p*‐value = 0.001), gastric cancer (*p*‐value = 0.001), and esophageal cancer (*p*‐value = 0.001)
Breitling, LP (2011) [40]	Germany (industry workers' database)	Prospective cohort of 19,090 men aged 25–64 years occupied in industry	GGT analyzed as quintiles; First quintile used as the reference group for the rest of the quintiles	16–22	Highest GGT quintile (GGT > 39) is associated with higher hazard for all‐cause cancer mortality, compared to the reference group (GGT < 11) (HR = 2.09; 95% CI = 1.60–2.74). Cancer incidence is not reported
Calori, G (2011) [41]	Italy (Cremona Study)	2074 randomly selected subjects aged 40 or more with Caucasian ethnicity	GGT analyzed as a continuous variable	15	Baseline serum GGT levels are significantly correlated to all‐cancer mortality within 15‐year follow‐up period. (HR = 1.002; 95% CI = 1.001–1.003) Cancer incidence is not reported
Cho, EJ (2023) [42]	South Korea (NHIS)	10,585,844 subjects aged 20 years or older	GGT analyzed as tertiles; first tertile used as the reference group for the rest of the tertiles	8.3	Highest GGT tertile (GGT > 44) correlates with higher cancer mortality hazard ratio (women: HR = 1.42; 95% CI = 1.40–1.44—men: HR = 1.60; 95% CI = 1.58–1.62)
Choi, YJ (2017) [43]	South Korea (NHIS)	8,388,256 subjects aged 40 years or older without prior cancer diagnosis, serious comorbidities, or viral hepatitis	GGT analyzed as quartiles; first quartile used as the reference group for the rest of the quartiles	8.7	Highest GGT quartile (GGT > 40) correlates with higher incidence of esophageal cancer (HR = 2.26; 95% CI = 2.07–2.45). 3rd quartile (24 < GGT < 39) correlates significantly with incidence of esophageal cancer (HR = 1.24; 95% CI = 1.14–1.36)
Grecian, S (2020) [44]	Scotland (E2DS)	1066 randomly chosen type 2 diabetes patients from E2DS prospective cohort	Subjects were categorized into two groups (normal vs. high GGT) (GGT > 55)	11	GGT is associated with HCC and cirrhosis incidence in diabetic patients.(OR = 3.55; 95% CI = 2.66–4.86) GGT is associated with HCC and cirrhosis incidence if added to FIB‐4 and APRI indexes
He, M (2021) [45]	United Kingdom (BioBank)	375,693 subjects aged 37–73 years without prior cancer diagnosis	GGT analyzed as deciles; first decile used as the reference group for the rest of the deciles	9	Increasing GGT is associated with lower colorectal cancer incidence (*p*‐value for trend = 0.001)
Hong, SW (2021) [46]	South Korea (NHIS)	8,120,665 adult subjects without prior cancer diagnosis	GGT analyzed as quartiles; First quartile used as the reference group for the rest of the quartiles	12	New‐onset esophageal cancer is associated with higher GGT levels; this association is significant for Q2 (HR = 1.22; 95% CI = 1.01–1.35), Q3 (HR = 1.445; 95% CI = 1.305–1.559), and Q4 (2.408; 95% CI = 2.184–2.654) GGT association with stomach cancer incidence is significant for Q3 (HR = 1.04; 95% CI = 1.02–1.07) and Q4 (HR = 1.121; 95% CI = 1.093–1.149). GGT association with Colorectal cancer incidence is significant for Q2 (HR = 1.03; 95% CI = 1.01–1.05), Q3 (HR = 1.09; 95% CI = 1.06–1.12), and Q4 (HR = 1.18; 95% CI = 1.16–1.21)
Hu, G (2008) [47]	Finland (multicentered)	62,015 subjects aged 25–64 years who attended to seven cross sectional studies across 30 years	GGT analyzed as quartiles; first quartile used as the reference group for the rest of the quartiles	30	Baseline GGT level is associated with liver cancers; The association is significant for Q2 (HR = 1.22; 95% CI = 1.01–1.35), Q3 (HR = 1.445; 95% CI = 1.305–1.559), and Q4 (2.408; 95% CI = 2.184–2.654) Coffee consumption and GGT levels show inverse interaction regarding liver cancer incidence
Ishiguro, S (2009) [48]	Japan (JPHC)	68,980 participants of a prospective cohort aged 40–69 years at entry	subjects were categorized into two groups (normal vs. high GGT) (GGT > 60)	12	High GGT participants (GGT > 60) had higher HCC incidence (HR = 5.5; 95% CI = 3.5–8.0)
Johansen, D (2009) [49]	Sweden (Malmö)	33,346 middle‐aged subjects with mean age of 50 years at entry	GGT analyzed as quartiles; first quartile used as the reference group for the rest of the quartiles	22.1	Pancreatic cancer showed a significant relative risk factor with increasing baseline GGT levels; Relative risk was significant in Q2 (RR = 1.83; 95% CI = 1.04–3.21) and Q4 (RR = 2.22; 95% CI = 1.24–4.04)
Katzke, V (2020) [50]	Europe (EPIC‐Heidelberg)	25,546 subjects from a multicentered European cohort aged 35–70 years	GGT analyzed as quartiles; first quartile used as the reference group for the rest of the quartiles	24–32	Baseline GGT levels are not significantly correlated to colorectal cancer after multivariate adjustment for BMI, alcohol use, smoking history, socioeconomics, and physical activity
Lee, CH (2021) [51]	South Korea (NHIS)	3,559,109 participants from national health registry of South Korea	4 consecutive GGT measurements during 4 years; GGT levels were categorized for each measurement. A cumulative score was assigned to each patients, and the score was analyzed as quartiles; first quartile used as the reference	6.8	Patients with highest cumulative GGT levels (4 scores) had a significant risk of digestive disease cancers. (HR = 1.88; 95% CI = 1.83–1.94). digestive disease cancers incidence were also significant for score 1 (HR = 1.28;95% CI = 1.35–1.46), score 2 (HR = 1.40;95% CI = 1.35–1.43), and score 3 (HR = 1.52;95% CI = 1.48–1.58). The trend of correlations was significant (*p*‐value < 0.001)
Liao, W (2023) [52]	United Kingdom (BioBank)	421,032 participants aged 40–69 with European ancestry	GGT was modeled both as a continuous scale and as quartiles; first quartile used as the reference	7.2	Compared to lowest GGT percentile group, Patients with highest GGT quartile had significantly higher risk of pancreatic cancer incidence both in men (HR = 1.72; 95% CI = 1.14–2.61) and women (HR = 1.75; 95% CI = 1.06–1.88)
Loh, WJ (2010) [53]	United Kingdom (HDDRISC)	1016 adult males from a cohort that investigates risk factors of coronary heart disease	Patients were assessed primarily for insulin‐resistance levels, along with metabolic serum biomarkers. GGT was modeled as a continuous variable and reported only for univariate Cox survival analysis of cancer mortality. (cancer incidence not assessed)	21.5	Univariate cox analysis for cancer‐related death showed a significant association between GGT levels (as a continuous variable) and cancer incidence (HR = 1.40; 95% CI = 1.02–1.94; *p*‐value = 0.04)
Mok, Y (2016) [13]	South Korea (KCPS)	1,662,087 participants aged 20–95 years with mean age of 41.1 years	GGT analyzed as quintiles; First quintile used as the reference group for the rest of the quartiles	17	Highest GGT quintile (GGT > 60) was associated with higher hazard for all cancers in both men (HR = 6.67; 95% CI = 5.88–7.57) and women(HR = 7.75; 95% CI = 6.41–8.94) Compared to the first GGT quintile, all other quantiles had significantly higher cancer rates in men. Cancer incidence trend with increasing GGT levels was significant for esophageal, laryngeal, gastric, colorectal, liver, bile duct, and pancreas cancers in men and liver cancer for women
Pons, M (2022) [54]	Spain (single‐centered)	Retrospective cohort analysis consisted of 996 NAFLD patients	GGT was modeled as a continuous scale	5	Median baseline GGT for NAFLD patients who later developed hepatocellular cancer (HCC; median = 187; range = 91–323) was significantly higher than non‐HCC participants.(median = 63; range = 36–114)
Ryu, S (2016) [55]	South Korea (KSHS)	396,720 participants aged more than 18 years that underwent health checkup	Participants were primary assessed to evaluate the association between baseline gallstone presence and gallbladder cancer incidence later on the course of the cohort	5.4	Patients with Hepatocellular carcinoma had significantly higher baseline GGT levels before HCC incidence (GGT Median(range) for HCC group: 19(12–34); for non‐HCC group (52(28–112)), *p*‐value:> 0.001)
Si, WK (2016) [56]	South Korea (SNUBH)	A cohort of 3544 participants with type 2 diabetes.	Participants were screened for hepatocellular carcinoma risk factors. Risk factors were tested in derviation group and validation group. GGT was categorize into two groups	10	High GGT showed a significant correlation with hepatocellular carcinoma incidence in both derivation cohort (*n* = 2364, *p*‐value = 0.001) and validation cohort (*n* = 1180, *p*‐value = 0.005) High GGT (GGT > 40) has a hazard ratio of 6.36 (95% CI = 2.36,17.15; *p*‐value = 0.001) for hepatocellular cancer incidence
Stepien, M (2016) [57]	Europe (EPIC)	A nested case control study from a 520,000‐subject cohort that investigates lifestyle associations with chronic disorders	121 hepatocellular carcinoma and 131 gallbladder and bile tract cancer patients included in the study with 1:2 control ratio. GGT analyzed as quartiles; first quartile used as the reference group for the rest of the quartiles	7.5	Hepatocellular carcinoma incidence correlated significantly with GGT baseline levels in the 4th quartile (OR = 5.70; 95% CI = 1.99–16.31). odds ratio for hepatocellular carcinoma incidence was not significant for 2nd and 3rd quartiles (Q2: OR = 1.18; 95% CI = 0.35–4.02, Q3:OR = 1.14.; 95% CI = 0.33–4.03), and The *p*‐value for data trend was significant. (*p*‐trend < 0.0001)
Strasak, AM (2008) (1) [11]	Austria (VHM&PP)	94,628 females aged over 18 years without prior malignancy	GGT analyzed as quartiles; first quartile used as the reference group for the rest of the quartiles	13.5	Incidence of cancer of any type was increased in GGT quartiles compared to 1st quartile. Digestive system cancers showed a significant incidence increase in 4th quartile (HR = 1.57; 95% CI = 1.25–1.97) compared to reference group. *p*‐value for trend in GGT increase was significant (p for trend = 0.002)
Strasak, AM (2008) (2) [58]	Austria (VHM&PP)	79,279 males aged over 18 years without prior malignancy	GGT levels were inquired as continuous variables. Best‐fitting regression model applied to model various percentiles.	12.5	Elevated GGT shows a dose–response relationship with cancer incidence. Patients with GGT > 60 show a hazard ratio = 1.32 (95% CI = 1.28–1.36) compared to the reference group
Tsuboya, T (2012) [59]	Japan (Ohsaki)	15,031 adults aged 40–79 years from a health checkup registry	GGT analyzed as quartiles; first quartile used as the reference group for the rest of the quartiles	9	Patients with highest GGT quartile showed significantly higher rates of cancer incidence (HR = 1.28; 95% CI = 1.08–1.53). Hazard ratios for colorectal cancer incidence was significant with increasing GGT levels (p fro trend = 0.02). esophageal and pancreatic cancers showed increased hazard ratio in high GGT patients, but this hazard ratio failed to reach significance
Van Hemelrijck, M (2011) [12]	Sweden (AMORIS)	545,460 adults aged more than 20 years from the apolipo‐protein mortality cohort study.	GGT analyzed as quartiles; First quartile used as the reference group for the rest of the quartiles	12.3	Compared to the first quartile, all three quartiles showed significance for increased digestive cancer hazard ratio. (Q2: HR = 1.07; 95% CI = 1.04–1.09; Q4: HR=; 95% CI=; Q4: HR=; 95% CI=)
Zhu, Z (2023) [60]	China (multicentered)	588 patients included in a risk factor analysis of gallbladder carcinoma in patients with gallstones	GGT levels were inquired as continuous variables. Patients were categorized as internal test samples and external validation samples	—	Gallstone patients with gallbladder carcinoma showed significantly higher baseline GGT compared to gallstone patients without gallbladder carcinoma, prior to the cancer development (*p*‐value:< 0.001)

One notable study addressed GGT association with cancer incidence not only at baseline GGT level but also as a repeated measurement; Lee et al. constructed a scoring system that categorized subjects based on their GGT levels on four consecutive measurements over 4 years [51] (Table 1). After analyzing 3.5 million subjects, they concluded that patients with the highest average GGT had an 88% higher risk of digestive system cancer incidence (HR 1.88, 95% CI 1.83–1.94). Interestingly, they showed that patients with only one GGT measurement higher than the reference group had a 28% higher risk (HR 1.28, 95% CI 1.35–1.46) of cancer development over a period of 6.8 years.

#### 3.2.1 Meta‐Analysis

**FIGURE 2:**
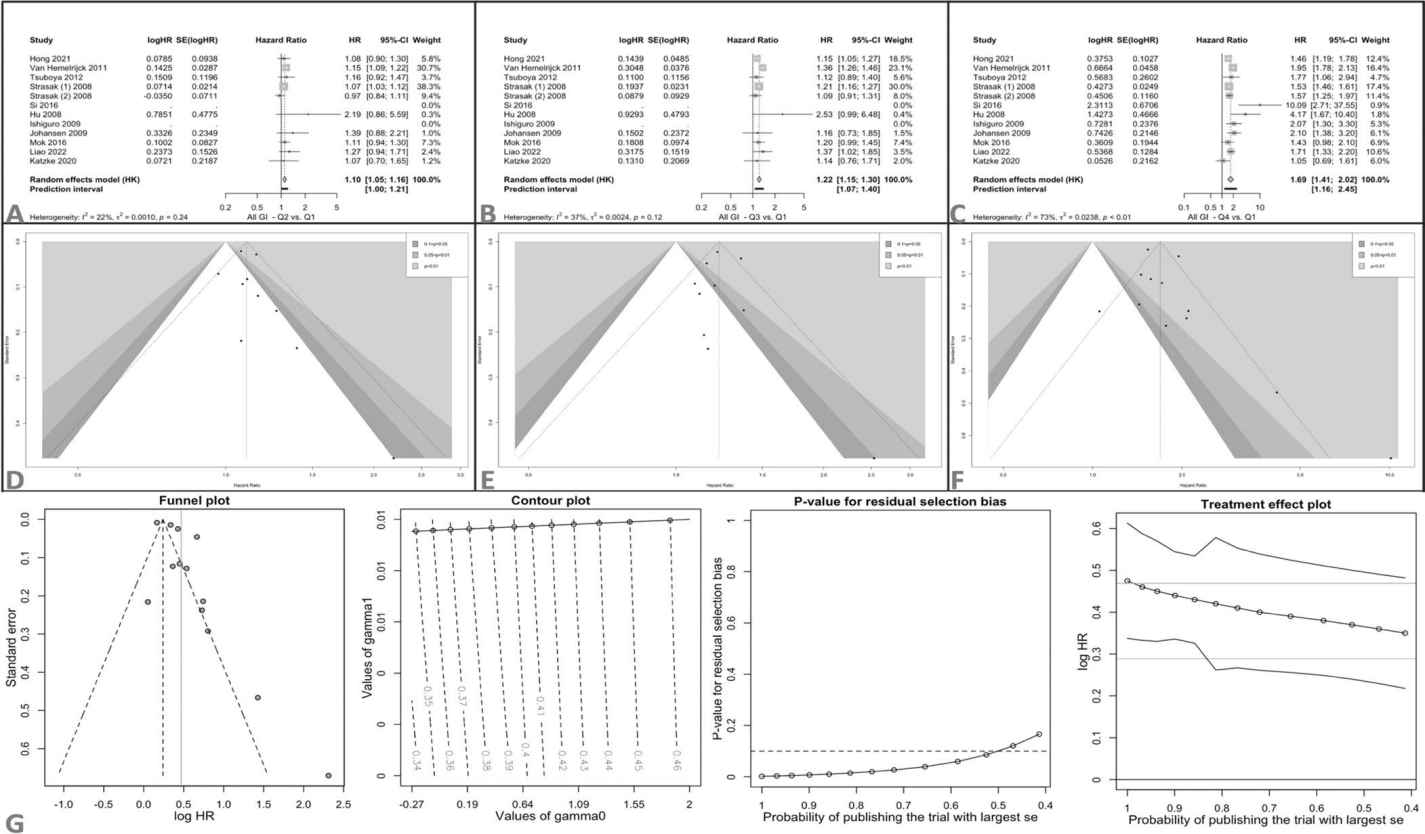
(A) Forest plot for meta‐analysis of Q2 versus Q1 of GGT effect on all GI cancers; (B) forest plot for meta‐analysis of Q3 versus Q1 of GGT effect on all GI cancers; (C) forest plot for meta‐analysis of Q4 versus Q1 of GGT effect on all GI cancers; (D) funnel plot for meta‐analysis A; (E) funnel plot for meta‐analysis B; (F) funnel plot for meta‐analysis C; and (G) Copas adjustment for meta‐analysis of Q4 versus Q1.

In meta‐analysis, compared to the Q1 group, Q4 patients had a 69% higher risk of digestive system cancer incidence over time (HR 1.69, 95% CI 1.41–2.02, *p* < 0.001), while patients in Q2 and Q3 had a 10% (HR 1.10, 95% CI 1.05–1.16, *p* = 0.002) and 22% (HR 1.22, 95% CI 1.15–1.30, *p* < 0.001) higher risk, respectively (Table 2, Figure 2A–C.).

**TABLE 2:** Pooled hazard ratios (HRs) for incidence gastrointestinal malignancies in subjects with 2nd quartile GGT levels in comparison to the reference group (1st Quartile).

		Studies (*n*)	Patients (*n*)	Pooled HR (95% CI)	*p*	Prediction interval	Heterogeneity and bias			Trim and fill	
							*I* ^2^	Egger P	Tau	HR (95% CI)	*p*
All GI		13	11,081,013								
	Q2			1.10 (1.05–1.16)	**0**.**002** *	1.00–1.21	22%	0.34	0.03	1.10 (1.04–1.16)	**0**.**005** *
	Q3			1.22 (1.15–1.30)	**< 0**.**001** *	1.07–1.41	37%	0.99	0.05	1.22 (1.15–1.30)	**< 0**.**001** *
	Q4			1.69 (1.41–2.02)	**< 0**.**001** *	1.16–2.45	73%	0.87	0.15	1.60 (1.30–1.97)	**0**.**002** *
Colon		5	10,368,789								
	Q2			1.03 (1.01–1.05)	**0**.**016** *	1.00–1.07	0%	0.61	0.00	1.03 (1.01–1.04)	**0**.**014** *
	Q3			1.09 (1.07–1.11)	**0**.**002** *	1.05–1.13	0%	0.47	0.00	1.09 (1.07–1.11)	**< 0**.**001** *
	Q4			1.18 (1.15–1.22)	**0**.**009** *	1.14–1.23	0%	0.57	0.00	1.18 (1.07–1.30)	**0**.**008** *
Esophagus		3	9,797,783								
	Q2			1.21 (1.11–1.33)	**0**.**010** *	0.62–2.37	0%	0.77	0.00	1.22 (1.11–1.33)	**0**.**010** *
	Q3			1.20 (0.69–2.10)	0.291	—^a^	70%	0.41	0.21	1.42 (0.94–2.15)	0.075
	Q4			1.72 (0.64–4.63)	0.142	—^a^	76%	0.34	0.41	2.41 (1.14–5.05)	**0**.**030** *
Liver		7	2,308,302								
	Q2			1.46 (1.34–1.58)	**0**.**002** *	1.23–1.74	0%	0.58	0.0–0.3	1.46 (1.34–1.58)	**0**.**001** *
	Q3			1.87 (1.55–2.26)	**0**.**001** *	1.32–2.64	16%	0.58	0.0–0.6	1.82 (1.57–2.11)	**0**.**001** *
	Q4			4.35 (2.62–7.23)	**0**.**004** *	0.98–19.31	79%	0.52	0.2–1.2	2.76 (1.57–4.84)	**0**.**002** *
Pancreas		5	2,676,956								
	Q2			1.13 (0.97–1.32)	0.097	0.88–1.44	0%	0.57	0.0–0.3	1.10 (0.95–1.28)	0.166
	Q3			1.17 (0.96–1.42)	0.099	0.91–1.49	0%	0.37	0.0–0.5	1.14 (0.95–1.39)	0.130
	Q4			1.57 (1.15–2.13)	**0**.**015** *	0.69–3.58	62%	0.25	0.0–0.7	1.22 (0.85–1.75)	0.226
Stomach		3	9,797,783								
	Q2			0.99 (0.36–2.72)	0.974	—;^a^	63%	0.86	0.0‐3.2	1.07 (0.44–2.62)	0.821
	Q3			1.04 (1.02–1.06)	**0**.**012** *	0.90–1.20	0%	0.74	0.0–0.2	1.04 (1.02–1.06)	**0**.**003** *
	Q4			1.12 (1.07–1.16)	**0**.**007** *	0.96–1.30	0%	0.23	0.0–0.3	1.12 (1.09–1.15)	**0**.**003** *
Bile duct^b^		1	1,662,087								
	Q2			1.18 (1.00–1.40)	0.908	—	—	—	—	—	—
	Q3			1.15 (0.97–1.35)	0.130	—	—	—	—	—	—
	Q4			1.37 (1.20–1.56)	**0**.**001** *	—	—	—	—	—	—

^a^ Prediction interval not calculable due to wide interval.

^b^ Only one study found.

* Significant at 0.05 level.

**FIGURE 3:**
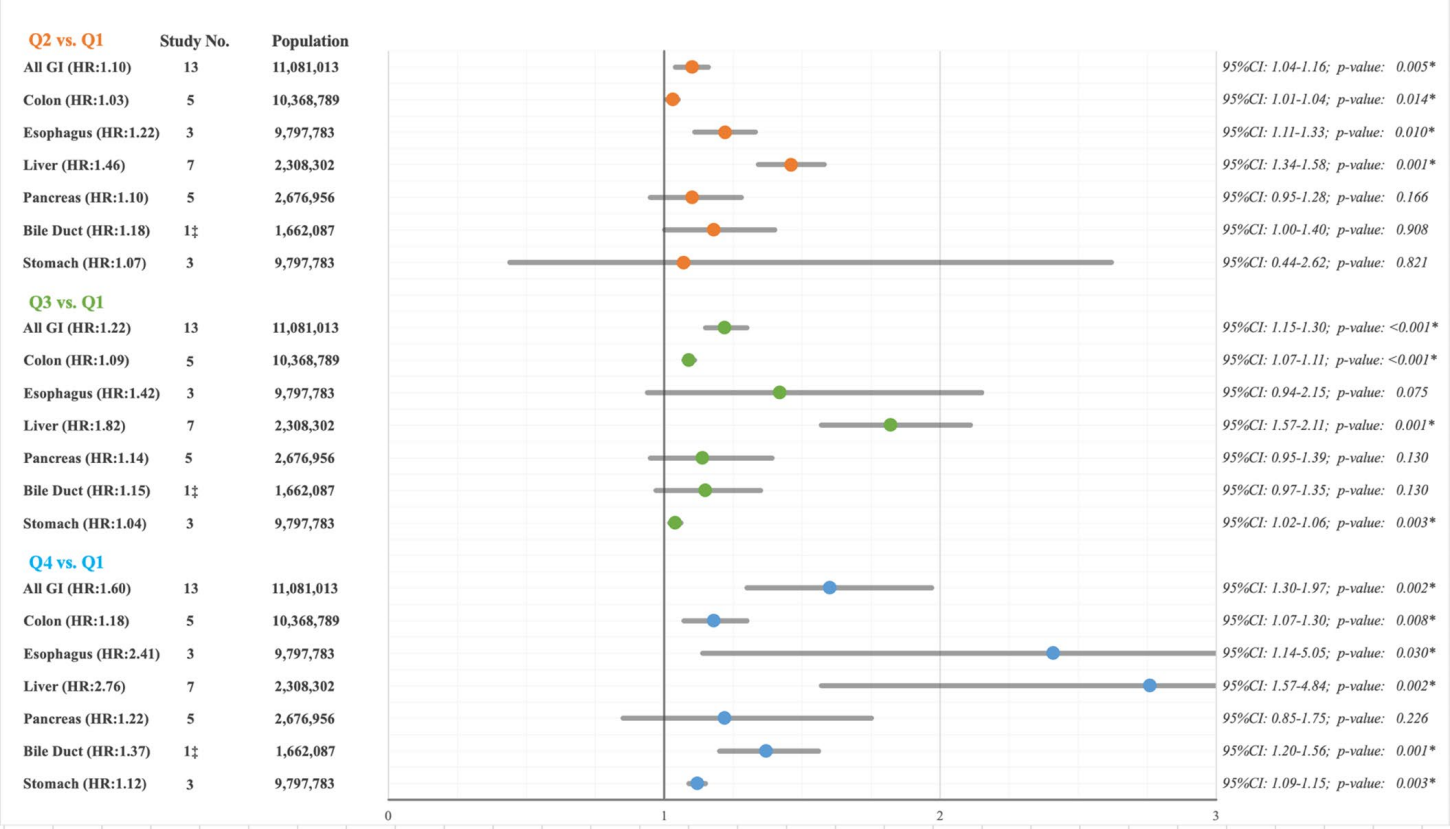
Summary of meta‐analyses for comparison of GGT quartiles in terms of GI cancer's incidence and their subgroups.

Figure 3 and Table 2 represent all analyses performed for all GI cancers, as well as each cancer type. GGT level quartiles also showed a significant trend for the HR of gastrointestinal cancers, implying a dose–response relation between GGT levels and the hazard for cancer incidence. A random‐effects prediction interval is significantly higher than one across all quartiles.

Egger's test resulted in no significant publication bias across any quartile. Trim and fill adjustment of HRs showed minimal change in the pooled HR, compared to the original analysis, further implying the robustness (Figure 2D–F and Table 2). The Copas adjustment for publication bias showed no bias in Q2 and Q4, while Q3 was a probable suspect (Figure 2G). The heterogeneity indexes were low in Q2 and Q3, and medium in Q4.

### 3.3 Colon and Rectum Cancer

We found seven studies addressing GGT levels in association with colorectal cancer incidence. Five studies reported HRs by categorizing GGT levels as quartiles [12, 13, 46, 50, 59], one as a binominal variable (low (< 55) vs. high) [45], and one as a categorical variable in a nested case–control study [39]. Overall, four studies reported significantly higher colorectal cancer incidence, while the other three failed to reach significance.

#### 3.3.1 Meta‐Analysis

The meta‐analysis established that compared to Q1, higher GGT levels showed a significantly increased risk of colorectal cancer incidence (Table 2, Figures S2–S4). Patients with GGT levels within Q4 had an 18% higher hazard ratio for new‐onset colorectal cancer over time (HR 1.18, 95% CI 1.15–1.22, *p* = 0.009). Q2 and Q3 levels of GGT showed similar significant effect sizes, compared with Q1 (Table 2). No heterogeneity or publication bias was observed in this analysis, and the trim and fill adjustment showed similar HRs to the original analysis. Finally, GGT levels higher than baseline showed a dose–response correlation with the onset of colorectal cancer.

### 3.4 Esophageal Cancer

Five studies sought to clarify the association of GGT with new‐onset esophageal cancer, including one nested case–control study and 4 cohort studies [13, 39, 43, 46, 59]. The nested case–control study reported a significant association between baseline GGT and esophageal cancer incidence for GGT levels categorized into quartiles (*p* = 0.001). The rest of the studies reported HRs ranging from 1.17 to 2.4; two of which were significant [43, 46]. It is worth mentioning that these two were reported from the same database (NHIS‐South Korea), and our meta‐analysis only included the study with higher‐quality appraisal results.

#### 3.4.1 Meta‐Analysis

The meta‐analysis subgroup for esophageal cancer showed a 21%, 20%, and 72% percent higher hazard of esophageal cancer incidence for Q2, Q3, and Q4 subjects, respectively (Table 2, Figures S5–S7). However, only the pooled HR of Q2 was significant (HR 1.21, 95% CI 1.11–1.33, *p*‐value = 0.01; Table 2) Following the Trim and fill adjustment for HRs, Q2, Q3, and Q4 showed 22%, 42%, and 141% higher risk of cancer development over time, with Q2 and Q4 values being significant (Q2: *p*‐value = 0.01; Q4: *p*‐value = 0.03). The heterogeneity level among Q2‐, Q3‐, and Q4‐included articles was low, moderate, and high, respectively (Table 2).

### 3.5 Liver Cancer

Nine studies investigated liver cancer incidence with varying GGT measurements [12, 13, 38, 44, 47, 48, 56, 57, 59], including one dose–response study that resulted in an HR of 2.4 (95% CI 2.19–2.65) if adjusted for high ALT, AST, ALP, BS, and TG [38]. However, no study provided comprehensive evidence to establish a clear dose–response relationship between serum GGT levels and liver cancer incidence. The remaining studies categorized patients into quartiles, all of which showed significantly higher liver cancer rates among Q4 individuals. Q2 and Q3 individuals had significantly higher hepatocellular carcinoma (HCC) rates in two studies [12, 13, 38, 44, 47, 48, 56, 57, 59].

#### 3.5.1 Meta‐Analysis

According to meta‐analysis, HCC incidence significantly correlated with patients' baseline GGT levels across all quartiles. HCC showed the steepest trend for correlation with GGT levels among the investigated cancers, with Q4 subjects having a 4.35 times higher risk of cancer incidence compared to the reference Q1 group (HR 4.35, 95% CI 2.62–7.23, *p*‐value = 0.004, Table 2 and Figures S8–S10). The pooled survival analysis remained significant following trim and fill adjustment, with Q2, Q3, and Q4 cases being 46%, 82%, and 176% more prone to liver cancer incidence over time, compared to the Q1 subjects.

### 3.6 Pancreas Cancer

Five studies included results specifically for pancreatic cancer incidence, two of which showed significantly higher incidence among Q4, one study among Q3, and no studies among Q2 subjects [12, 13, 49, 52, 59].

#### 3.6.1 Meta‐Analysis

Our random‐effects meta‐analysis for pancreatic cancer incidence showed that Q4 individuals are at 57% higher risk of cancer development compared to the reference group (HR 1.57, 95% CI 1.15–2.13, *p*‐value = 0.015). Q2 and Q3 patients also showed a higher risk of pancreatic cancer development. Nevertheless, these findings failed to reach statistical significance (Table 2 and Figures S11–S13).

### 3.7 Gastric Cancer

Three studies were included in qualitative synthesis, all of which modeled gastric cancer incidence as a Cox survival analysis model across GGT quartiles [13, 46, 59]. The largest study among all, investigated 8.1 million subjects over 12 years and showed an HR of 1.12 (95% CI 1.09–1.15) for stomach cancer incidence among Q4 subjects compared to Q1 subjects. The other two studies were in line with this finding; nevertheless, they failed to attain statistical significance.

#### 3.7.1 Meta‐Analysis

Gastric cancer incidence showed a modest but significant trend of association with increasing GGT levels. Out of 9.7 million subjects included in the pooled study, unlike the Q2 group, Q3 and Q4 subjects showed a 4% (HR 1.04, 95% CI 1.02–1.06, *p*‐value = 0.012) and 12% (HR 1.12, 95% CI 1.07–1.16, *p*‐value = 0.007) higher cancer incidence compared to the reference group (Table 2 and Figures S14–S16). The meta‐analysis of all quartiles showed minimal heterogeneity, and the trim‐and‐fill method for publication bias showed similar HRs to the uncorrected model (*p*‐value = 0.003).

### 3.8 Bile Duct Cancers

Only two articles addressed gallbladder and bile duct cancers across the GGT levels spectrum, including a cohort of 1.6 million participants over 17 years of follow‐up. The latter study showed that subjects with Q4 GGT levels, had a 1.2‐fold (95% CI 0.99–1.52) and 1.6 (95% CI 1.24–2.07) higher hazard for gallbladder cancer in men and women, respectively. The other study was conducted on patients with gallstones in a case–control setting and found patients with gallstones who had higher GGT levels had a greater risk of gallbladder cancer development.

## 4 Discussion

To the best of our knowledge, this is the first systematic review and meta‐analysis of GGT levels' association with the incidence of GI cancers. By inclusion of 26 studies, we established that GGT predicts the incidence of colon, esophagus, liver, stomach, and overall digestive cancers (Figure 3). An increase in GGT by 10 units raised the risk of colorectal, esophageal, and hepatocellular cancers by 3%, 21%, and 46%, respectively. Other digestive cancers also show a higher incidence in patients with elevated GGT levels. Notably, pooled analysis demonstrates GGT's role as a risk factor for stomach cancer, a crucial finding due to its high prevalence, insidious onset, and poor prognosis. Therefore, GGT should not be viewed like other liver biomarkers, but as a predictive marker for systemic inflammatory pathways, including cancer. The findings of the current review not only provide researchers with valuable data for future research but also informs clinical practice when GGT levels are persistently raised in the absence of liver disease.

Our qualitative analysis highlighted a large cohort study that demonstrated the predictive value of GGT in longitudinal assessments [51]. After following 3.5 million subjects for 6.8 years, patients with consecutively elevated GGT had an HR of 1.88 for GI cancers compared to the reference group. This finding emphasizes the importance of GGT as a widely available, non‐invasive predictive test for systemic inflammatory disorders including cancer. Our meta‐analysis also revealed a similar result. Notably, the correlation between GGT levels and the incidence of GI cancers differed with the site of cancer. The highest risk was for esophageal cancer and liver cancer while the lowest effect size was observed for gastric and colorectal cancers.

**FIGURE 4:**
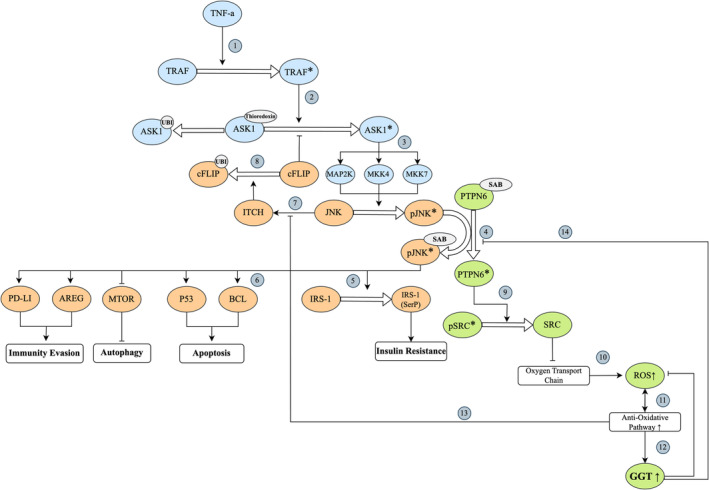
Molecular association of GGT elevation, insulin resistance, and cancer incidence. (1) Systemic inflammation, marked by TNF‐a elevation, activates TRAF [67, 68]. (2) TRAF activates ASK1 by dissociating it from thioredoxin [85, 86]. (3) Activated ASK1 induces MAP2K, MKK4, and MKK7 to activate JNK by phosphorylation [66, 69]. (4) pJNK binds to mitochondrial scaffold SAB and is stabilized in its active form. SAB dissociates from PTPN6 to attach to pJNK [69, 70, 71, 72]. (5) pJNK phosphorylates IRS‐1 at serine residues, turning IRS‐1 resistant to serum insulin levels [67, 73]. (6) pJNK also induces apoptosis, autophagy, and immunity evasion by activating P53. BCL, AREG, PD‐LI, and inhibiting mTOR [73, 74]. (7) JNK also activates ITCH, a ligase that degrades cFLIP by ubiquitination [69, 73, 75, 76]. (8) cFLIP is an antiapoptotic protein that inhibits ASK1 activation. Conclusively, Activation of ITCH by JNK leads to positive feedback that further increases the activated JNK level [69, 73, 75, 76]. (9) As SAB dissociates from PTPN6 to bind to JNK, the freed PTPN6 dephosphorylates SRC from its active form [77, 78, 79, 80]. (10) SRC, when active, protects mitochondrial respiration by stabilizing the oxygen transport chain. Inactive SRC leads to transport chain dissociation and ROS elevation [77, 78, 79, 80]. (11) High ROS levels induce elevation of anti‐oxidative pathways, and. (12) GGT rises as a consequence [81, 82, 83, 84]. (13) Anti‐oxidative pathways also inhibit ITCH activation, thereby acting as negative feedback by inhibiting ASK‐1 and JNK activation [75, 76].

Several studies have sought to determine if GGT is directly associated with cancer pathogenesis or is merely a consequence of cancer‐cell transformation. Studies on insulin resistance have shown that high GGT coincides with an increase in inflammatory interleukins and tumor necrosis factor‐alpha (TNF‐ α) [61, 62]. Consequently, TNF‐α inactivates insulin receptor substrate 1 (IRS‐1) mediated by c‐Jun N‐terminal kinase (JNK) [63, 64, 65, 66] (Figure 4). Activated JNK (pJNK) frees mitochondrial scaffold protein SAB from protein tyrosine phosphatase non‐receptor 6 (PTPN6) to stay stabilized in its active state [69]. Following stabilization, p‐JNK induces insulin resistance, apoptosis, autophagy, and immunity evasion via IRS‐1, P53/BCL, MTOR, and PD‐LI/amphiregulin, respectively [73]. pJNK‐SAB bonding unleashes PTPN6 from SAB and allows it to inhibit SRC, a stabilizer of mitochondrial respiration. Inhibition of SRC disrupts the electron transport chain, leading to an excess of reactive oxygen species (ROS) [69]. In response, GGT will rise in cells either as a protector of the cell to stop ROS damage via the glutathione pathway or as a byproduct of the disrupted JNK pathway [77, 78, 79, 80, 85]. GGT has also been shown to counteract JNK by inactivating it from the stabilized state since it has been shown that inhibiting GGT results in sustained JNK activity [69, 86, 87]. These phenomena are in line with our question of how elevated GGT may be associated with insulin resistance and cancer incidence. One can conclude that any malfunction in the pathway, such as the widely known P53 knockout mutations, may disrupt the equation for JNK's ambivalent nature as a tumor‐suppressor and proto‐oncogene, and could eventually lead to malignancy. It is noteworthy that a cell's response to activated JNK may differ based on the tissue inflammatory state. Exposing normal cells to activated JNK results in highly productive cell metabolism, whereas cells in a pro‐inflammatory state would not show better metabolic outcomes, but only higher ROS production [69, 87, 88, 89]. In sum, an inflammatory state induces a cellular pathway mediated by JNK that leads to oxidative stress, and mitochondrial disruption, cellular‐immunity evasion, and GGT elevation.

Our findings have several clinical and research implications. While an association was observed, GGT's use as a surveillance test is questioned due to its nonspecific nature. Hence, further studies that determine its sensitivity, specificity, and negative/positive predictive values (NPV and PPV) for cancer diagnosis are warranted. Further, GGT specificity for GI cancer type needs further exploration. Due to its association with a higher risk of GI cancer incidence, clinicians should not ignore high GGT levels, let alone the other conditions related to high GGT levels such as cardiovascular diseases [90].

Several studies have suggested that different populations have various GGT levels based on ethnicity, culture, and food preferences [3]. There have also been several reports that digestive tract cancers show widely varying incidence and prevalence in different regions of the world. For instance, different subtypes of esophageal cancers prevail in European regions, the Middle East, and Southeast Asia [91, 92, 93, 94, 95]. Future literature may answer whether these variations in GGT in different ethnicities are in line with variations in cancer incidence in those ethnicities. In order to minimize the effect of GGT variations across different populations in different cohorts, we applied a standardized treatment effect and random‐effect model to our meta‐analysis [96]. We have also applied a random‐effects model to address other unknown parameters that may affect the normal GGT range in a population. It is noteworthy that all cohorts included in this study comprised large groups of subjects, which further minimizes the risk of a small‐study effect in a random‐effects model and the need for subgrouping the population for multivariable analysis.

This review found several gaps in the current literature and some limitations. Firstly, while HR is a suitable scale for defining the predictability of GGT, a stronger statistic such as a receiver operating characteristic (ROC) could further elucidate the accuracy of GGT as a predictor of digestive system cancers. Then, the low number of analyzed studies for some of the GI cancer subtypes might lead to limitations in the interpretation of these findings based on our findings. Future studies should be encouraged to report GGT both as quartiles and continuous measurements so that clinicians might gain insights into the dose–response relationships between cancer incidence and rising GGT levels.

## 5 Conclusions

In conclusion, this meta‐analysis has shown that even mild GGT elevations above the normal range are associated with increases in the risk of colon, esophagus, liver, and gastric cancers. We also show a positive trend of GGT levels with digestive cancer incidence after pooling 11 million subjects in the meta‐analysis. Our literature review on the molecular basis of cancer pathogenesis in oxidative stress demonstrates in detail that systemic inflammation triggers several key molecules such as JNK and ASK1 that are regulators of apoptosis, cancer‐cell immune evasion, insulin resistance, mitochondrial respiration, and oxidative stress, all of which are associated with GGT levels.

## Author Contributions

**Alireza Ramandi:** conceptualization (equal), data curation (equal), formal analysis (equal), funding acquisition (equal), investigation (equal), methodology (equal), project administration (equal), software (equal), validation (equal), visualization (equal), writing – original draft (equal). **Jacob George:** conceptualization (equal), formal analysis (equal), investigation (equal), supervision (equal), validation (equal), writing – original draft (equal), writing – review and editing (equal). **Amir Hossein Behnoush:** data curation (equal), formal analysis (equal), methodology (equal), project administration (equal), visualization (equal), writing – original draft (equal). **Alireza Delavari:** formal analysis (equal), validation (equal), writing – review and editing (equal). **Zahra Mohammadi:** methodology (equal), software (equal), writing – review and editing (equal). **Hossein Poustchi:** conceptualization (equal), data curation (equal), formal analysis (equal), investigation (equal), methodology (equal), resources (equal), software (equal), supervision (equal), validation (equal), writing – review and editing (equal). **Reza Malekzadeh:** conceptualization (equal), investigation (equal), methodology (equal), supervision (equal), validation (equal), writing – review and editing (equal).

## Ethics Statement

The study protocol was approved by the review board of the Digestive Diseases Research Institute of Tehran University of Medical Sciences and ethics committees at Tehran University of Medical Sciences (IR.TUMS.MEDICINE.REC.1401.701).

## Consent

The authors have nothing to report.

## Conflicts of Interest

The authors declare no conflicts of interest.

## Supporting information


Data S1:

## Data Availability

The authors have nothing to report.
