# Supplementary material for: The Association Between Serum Gamma‐Glutamyl Transferase and Gastrointestinal Cancer Risk: A Systematic Review and Meta‐Analysis
Source: Cancer Med. 2025 Jan 16;14(2):e70581. doi: 10.1002/cam4.70581 (PMC11736428; doi:10.1002/cam4.70581)
Supplement: Supplementary file 1 — Data S1: [file CAM4-14-e70581-s001.docx]

**Supplementary Materials**

***Supplementary Table 1.*** *The search queries used for each database and the search results*

| **Query** | | **Results (No.)**  **(May 27, 2024)** |
| --- | --- | --- |
| **PubMed** | | |
| #1 | ("gamma-Glutamyltransferase"[Mesh] OR “gamma Glutamyltransferase” OR “GGTP” OR “gammaglutamyltransferase” OR “gamma-Glutamyl Transpeptidase” OR “Transpeptidase, gamma-Glutamyl” OR “Transpeptidase, gamma-Glutamyl” OR “gamma Glutamyl Transpeptidase” OR “Glutamyl Transpeptidase” OR “Transpeptidase, Glutamyl”) | 20,740 |
| #2 | ("Mouth Neoplasms"[Mesh] OR “Oropharyngeal Neoplasms"[Mesh] OR “Stomach Neoplasms"[Mesh] OR "Intestinal Neoplasms"[Mesh] OR “Cecal Neoplasms"[Mesh] OR "Colorectal Neoplasms"[Mesh] OR "Immunoproliferative Small Intestinal Disease"[Mesh] OR "Jejunal Neoplasms"[Mesh] OR "Zollinger-Ellison Syndrome"[Mesh] OR "Digestive System Neoplasms"[Mesh] OR "Biliary Tract Neoplasms"[Mesh] OR "Liver Neoplasms"[Mesh] OR "Pancreatic Neoplasms"[Mesh] OR "Peritoneal Neoplasms"[Mesh] OR “mouth cancer” OR “mouth cancer” OR “cancer, mouth” OR “intraoral cancer” OR “mouth cancer” OR “mouth mucosa cancer” OR “oral cancer” OR “oral cavity cancer” OR “oropharynx cancer” OR “oropharynx cancer” OR “cancer, oropharyngeal” OR “oropharyngeal cancer” OR “oropharynx cancer” OR “esophagus cancer” OR “esophagus cancer” OR “cancer of the abdominal esophagus” OR “cancer of the cervical esophagus” OR “cancer of the cervical oesophagus” OR “cancer of the esophagus” OR “cancer of the oesophagus” OR “cancer of the thoracic esophagus” OR “cancer of the thoracic oesophagus” OR “cancer, esophagus” OR “carcinogenesis in the esophagus” OR “esophageal cancer” OR “esophageal carcinogenesis” OR “esophageal malignancies” OR “esophageal malignancy” OR “esophagus cancer” OR “malignancies of the esophagus” OR “malignancies of the oesophagus” OR “malignancy of the esophagus” OR “malignancy of the oesophagus” OR “malignant esophageal disease” OR “malignant esophageal tumor” OR “malignant neoplasm of the esophagus” OR “malignant neoplasm of the oesophagus” OR “malignant oesophageal disease” OR “malignant tumor of the esophagus” OR “oesophageal cancer” OR “oesophageal carcinogenesis” OR “oesophageal malignancies” OR “oesophageal malignancy” OR “oesophagus cancer” OR “stomach cancer” OR “stomach cancer” OR “cancer of the cardia” OR “cancer of the gastric antrum” OR “cancer of the gastric body” OR “cancer of the gastric cardia” OR “cancer of the gastric fundus” OR “cancer, stomach” OR “cardia cancer” OR “gastric antral cancer” OR “gastric antrum cancer” OR “gastric body cancer” OR “gastric cancer” OR “gastric cardia cancer” OR “gastric cardiac cancer” OR “gastric malignancies” OR “gastric malignancy” OR “malignancies of the stomach” OR “malignancy of the stomach” OR “malignant gastric neoplasm” OR “malignant gastric tumor” OR “malignant neoplasm of the stomach” OR “malignant neoplasms of the stomach” OR “malignant tumor of the stomach” OR “malignant tumors of the stomach” OR “malignant tumour of the stomach” OR “malignant tumours of the stomach” OR “pyloric cancer” OR “stomach cancer” OR “stomach malignancies” OR “stomach malignancy” OR “intestine cancer” OR “intestine cancer” OR “bowel cancer” OR “cancer of the bowel” OR “cancer of the intestine” OR “cancer, intestine” OR “gut cancer” OR “gut carcinogenesis” OR “gut malignancy” OR “intestinal cancer” OR “intestinal cancerogenesis” OR “intestinal carcinogenesis” OR “intestinal malignancies” OR “intestinal malignancy” OR “intestine cancer” OR “intestine carcinogenesis” OR “intestine malignancies” OR “intestine malignancy” OR “malignant intestinal neoplasm” OR “duodenum cancer” OR “duodenum cancer” OR “cancer of the duodenal” OR “cancer of the duodenum” OR “duodenal cancer” OR “duodenal carcinogenesis” OR “duodenal malignancies” OR “duodenal malignancy” OR “duodenum cancer” OR “jejunum cancer” OR “jejunum cancer” OR “cancer of the jejunum” OR “cancer, jejunum” OR “jejunal cancer” OR “jejunal carcinogenesis” OR “jejunal malignancy” OR “jejunum cancer” OR “malignant tumor of the jejunum” OR “ileum cancer” OR “ileum cancer” OR “cancer of the ileum” OR “cancer, ileum” OR “ileal cancer” OR “ileum cancer” OR “malignant ileal” OR “cecum cancer” OR “cecum cancer” OR “caecal cancer” OR “caecum cancer” OR “cancer of the cecum” OR “cancer, cecum” OR “cecal cancer” OR “cecum cancer” OR “cecum sarcoma” OR “colorectal cancer” OR “colorectal cancer” OR “cancer of colon and rectum” OR “cancer of rectum and colon” OR “cancer of the colon and rectum” OR “cancer of the colon and the rectum” OR “cancer of the rectum and colon” OR “cancer of the rectum and the colon” OR “colo-rectal cancer” OR “colo-rectal carcinogenesis” OR “colo-rectal malignancies” OR “colo-rectal malignancy” OR “colorectal cancer” OR “colorectal cancerogenesis” OR “colorectal carcinogenesis” OR “colorectal malignancies” OR “colorectal malignancy” OR “malignancies of the colon and rectum” OR “malignancy of colon and rectum” OR “malignancy of the colon and rectum” OR “recto-colonic cancer” OR “rectocolonic cancer” OR “liver cancer” OR “liver cancer” OR “cancer of the liver” OR “cancer, liver” OR “carcinomatous liver” OR “hepatic cancer” OR “hepatic malignancies” OR “hepatic malignancy” OR “hepatocellular cancer” OR “hepatocellular carcinomatosis” OR “hepatocellular malignancies” OR “hepatocellular malignancy” OR “hepatocyte cancer” OR “liver cancer” OR “liver cell cancer” OR “liver malignancies” OR “liver malignancy” OR “liver primary cancer” OR “malignancies of the liver” OR “malignancy of the liver” OR “malignant hepatic neoplasm” OR “malignant hepatic tumor” OR “malignant hepatic tumour” OR “malignant liver neoplasm” OR “malignant liver tumor” OR “malignant liver tumour” OR “malignant neoplasm of the liver” OR “malignant neoplasms of the liver” OR “malignant tumor of the liver” OR “malignant tumors of the liver” OR “malignant tumour of the liver” OR “malignant tumours of the liver” OR “primary liver cancer” OR “biliary tract cancer” OR “biliary tract cancer” OR “biliary cancer” OR “biliary carcinogenesis” OR “biliary malignancies” OR “biliary malignancy” OR “biliary system cancer” OR “biliary tract cancer” OR “cancer of the biliary system” OR “cancer of the biliary tract” OR “malignancies of the biliary tract” OR “malignancy of the biliary tract” OR “malignant tumor of biliary tract” OR “pancreas cancer” OR “pancreas cancer” OR “carcinogenesis in the pancreas” OR “carcinogenesis of the pancreas” OR “malignancies of the pancreas” OR “malignancy of the pancreas” OR “malignant neoplasm of the pancreas” OR “malignant pancreatic neoplasm” OR “malignant pancreatic tumor” OR “malignant pancreatic tumour” OR “malignant tumor of the pancreas” OR “malignant tumour of the pancreas” OR “pancreas cancer” OR “pancreas carcinogenesis” OR “pancreas malignancies” OR “pancreas malignancy” OR “pancreatic cancer” OR “pancreatic cancerogenesis” OR “pancreatic carcinogenesis” OR “pancreatic malignancies” OR “pancreatic malignancy” OR “gastrointestinal stromal tumor” OR “gastrointestinal stromal tumor” OR “gi stromal tumor” OR “gastric stromal sarcoma” OR “gastro-intestinal stromal tumor” OR “gastro-intestinal stromal tumour” OR “gastrointestinal autonomic nerve tumor” OR “gastrointestinal pacemaker cell tumor” OR “gastrointestinal stroma tumor” OR “gastrointestinal stroma tumour” OR “gastrointestinal stromal neoplasm” OR “gastrointestinal stromal sarcoma” OR “gastrointestinal stromal tumor” OR “gastrointestinal stromal tumors” OR “gastrointestinal stromal tumour” OR “gastrointestinal stromal tumours” OR “liver adenoma” OR “liver adenoma” OR “adenoma in the liver” OR “adenoma of the liver” OR “adenoma, liver cell” OR “benign hepatoma” OR “hepatic adenoma” OR “hepatocellular adenoma” OR “liver adenoma” OR “liver cell adenoma”) | 1,112,618 |
| #3 | #1 AND #2 | 2,539 |
| **Embase** | | |
| #1 | ('gamma glutamyltransferase'/exp OR 'gamma glutamyltransferase' OR '(5 glutamyl) peptide:amino acid 5 glutamyltransferase':ti,ab,kw OR '4 glutamyl transferase':ti,ab,kw OR 'alpha glutamyltranspeptidase':ti,ab,kw OR 'e.c. 2.3.2.2':ti,ab,kw OR 'gamma glutamyl transferase':ti,ab,kw OR 'gamma glutamyl transpeptidase':ti,ab,kw OR 'gamma glutamyltransferase':ti,ab,kw OR 'gamma glutamyltranspeptidase':ti,ab,kw OR 'gamma gt':ti,ab,kw OR 'gamma-glutamyltransferase':ti,ab,kw OR 'gammaglutamyl transpeptidase':ti,ab,kw OR 'glutamate transpeptidase':ti,ab,kw OR 'glutamic transferase':ti,ab,kw OR 'glutamyl transferase':ti,ab,kw OR 'glutamyl transpeptidase':ti,ab,kw OR 'glutamyltransferase':ti,ab,kw OR 'glutamyltranspeptidase':ti,ab,kw OR 'levo glutamyltranspeptidase':ti,ab,kw OR 'transpeptidase':ti,ab,kw) | 74,256 |
| #2 | ('mouth cancer'/exp OR 'mouth cancer' OR 'cancer, mouth':ti,ab,kw OR 'intraoral cancer':ti,ab,kw OR 'mouth cancer':ti,ab,kw OR 'mouth mucosa cancer':ti,ab,kw OR 'oral cancer':ti,ab,kw OR 'oral cavity cancer':ti,ab,kw OR 'oropharynx cancer'/exp OR 'oropharynx cancer' OR 'cancer, oropharyngeal':ti,ab,kw OR 'oropharyngeal cancer':ti,ab,kw OR 'oropharynx cancer':ti,ab,kw OR 'esophagus cancer'/exp OR 'esophagus cancer' OR 'cancer of the abdominal esophagus':ti,ab,kw OR 'cancer of the cervical esophagus':ti,ab,kw OR 'cancer of the cervical oesophagus':ti,ab,kw OR 'cancer of the esophagus':ti,ab,kw OR 'cancer of the oesophagus':ti,ab,kw OR 'cancer of the thoracic esophagus':ti,ab,kw OR 'cancer of the thoracic oesophagus':ti,ab,kw OR 'cancer, esophagus':ti,ab,kw OR 'carcinogenesis in the esophagus':ti,ab,kw OR 'esophageal cancer':ti,ab,kw OR 'esophageal carcinogenesis':ti,ab,kw OR 'esophageal malignancies':ti,ab,kw OR 'esophageal malignancy':ti,ab,kw OR 'esophagus cancer':ti,ab,kw OR 'malignancies of the esophagus':ti,ab,kw OR 'malignancies of the oesophagus':ti,ab,kw OR 'malignancy of the esophagus':ti,ab,kw OR 'malignancy of the oesophagus':ti,ab,kw OR 'malignant esophageal disease':ti,ab,kw OR 'malignant esophageal tumor':ti,ab,kw OR 'malignant neoplasm of the esophagus':ti,ab,kw OR 'malignant neoplasm of the oesophagus':ti,ab,kw OR 'malignant oesophageal disease':ti,ab,kw OR 'malignant tumor of the esophagus':ti,ab,kw OR 'oesophageal cancer':ti,ab,kw OR 'oesophageal carcinogenesis':ti,ab,kw OR 'oesophageal malignancies':ti,ab,kw OR 'oesophageal malignancy':ti,ab,kw OR 'oesophagus cancer':ti,ab,kw OR 'stomach cancer'/exp OR 'stomach cancer' OR 'cancer of the cardia':ti,ab,kw OR 'cancer of the gastric antrum':ti,ab,kw OR 'cancer of the gastric body':ti,ab,kw OR 'cancer of the gastric cardia':ti,ab,kw OR 'cancer of the gastric fundus':ti,ab,kw OR 'cancer, stomach':ti,ab,kw OR 'cardia cancer':ti,ab,kw OR 'gastric antral cancer':ti,ab,kw OR 'gastric antrum cancer':ti,ab,kw OR 'gastric body cancer':ti,ab,kw OR 'gastric cancer':ti,ab,kw OR 'gastric cardia cancer':ti,ab,kw OR 'gastric cardiac cancer':ti,ab,kw OR 'gastric malignancies':ti,ab,kw OR 'gastric malignancy':ti,ab,kw OR 'malignancies of the stomach':ti,ab,kw OR 'malignancy of the stomach':ti,ab,kw OR 'malignant gastric neoplasm':ti,ab,kw OR 'malignant gastric tumor':ti,ab,kw OR 'malignant neoplasm of the stomach':ti,ab,kw OR 'malignant neoplasms of the stomach':ti,ab,kw OR 'malignant tumor of the stomach':ti,ab,kw OR 'malignant tumors of the stomach':ti,ab,kw OR 'malignant tumour of the stomach':ti,ab,kw OR 'malignant tumours of the stomach':ti,ab,kw OR 'pyloric cancer':ti,ab,kw OR 'stomach cancer':ti,ab,kw OR 'stomach malignancies':ti,ab,kw OR 'stomach malignancy':ti,ab,kw OR 'intestine cancer'/exp OR 'intestine cancer' OR 'bowel cancer':ti,ab,kw OR 'cancer of the bowel':ti,ab,kw OR 'cancer of the intestine':ti,ab,kw OR 'cancer, intestine':ti,ab,kw OR 'gut cancer':ti,ab,kw OR 'gut carcinogenesis':ti,ab,kw OR 'gut malignancy':ti,ab,kw OR 'intestinal cancer':ti,ab,kw OR 'intestinal cancerogenesis':ti,ab,kw OR 'intestinal carcinogenesis':ti,ab,kw OR 'intestinal malignancies':ti,ab,kw OR 'intestinal malignancy':ti,ab,kw OR 'intestine cancer':ti,ab,kw OR 'intestine carcinogenesis':ti,ab,kw OR 'intestine malignancies':ti,ab,kw OR 'intestine malignancy':ti,ab,kw OR 'malignant intestinal neoplasm':ti,ab,kw OR 'duodenum cancer'/exp OR 'duodenum cancer' OR 'cancer of the duodenal':ti,ab,kw OR 'cancer of the duodenum':ti,ab,kw OR 'duodenal cancer':ti,ab,kw OR 'duodenal carcinogenesis':ti,ab,kw OR 'duodenal malignancies':ti,ab,kw OR 'duodenal malignancy':ti,ab,kw OR 'duodenum cancer':ti,ab,kw OR 'jejunum cancer'/exp OR 'jejunum cancer' OR 'cancer of the jejunum':ti,ab,kw OR 'cancer, jejunum':ti,ab,kw OR 'jejunal cancer':ti,ab,kw OR 'jejunal carcinogenesis':ti,ab,kw OR 'jejunal malignancy':ti,ab,kw OR 'jejunum cancer':ti,ab,kw OR 'malignant tumor of the jejunum':ti,ab,kw OR 'ileum cancer'/exp OR 'ileum cancer' OR 'cancer of the ileum':ti,ab,kw OR 'cancer, ileum':ti,ab,kw OR 'ileal cancer':ti,ab,kw OR 'ileum cancer':ti,ab,kw OR 'malignant ileal':ti,ab,kw OR 'cecum cancer'/exp OR 'cecum cancer' OR 'caecal cancer':ti,ab,kw OR 'caecum cancer':ti,ab,kw OR 'cancer of the cecum':ti,ab,kw OR 'cancer, cecum':ti,ab,kw OR 'cecal cancer':ti,ab,kw OR 'cecum cancer':ti,ab,kw OR 'cecum sarcoma':ti,ab,kw OR 'colorectal cancer'/exp OR 'colorectal cancer' OR 'cancer of colon and rectum':ti,ab,kw OR 'cancer of rectum and colon':ti,ab,kw OR 'cancer of the colon and rectum':ti,ab,kw OR 'cancer of the colon and the rectum':ti,ab,kw OR 'cancer of the rectum and colon':ti,ab,kw OR 'cancer of the rectum and the colon':ti,ab,kw OR 'colo-rectal cancer':ti,ab,kw OR 'colo-rectal carcinogenesis':ti,ab,kw OR 'colo-rectal malignancies':ti,ab,kw OR 'colo-rectal malignancy':ti,ab,kw OR 'colorectal cancer':ti,ab,kw OR 'colorectal cancerogenesis':ti,ab,kw OR 'colorectal carcinogenesis':ti,ab,kw OR 'colorectal malignancies':ti,ab,kw OR 'colorectal malignancy':ti,ab,kw OR 'malignancies of the colon and rectum':ti,ab,kw OR 'malignancy of colon and rectum':ti,ab,kw OR 'malignancy of the colon and rectum':ti,ab,kw OR 'recto-colonic cancer':ti,ab,kw OR 'rectocolonic cancer':ti,ab,kw OR 'liver cancer'/exp OR 'liver cancer' OR 'cancer of the liver':ti,ab,kw OR 'cancer, liver':ti,ab,kw OR 'carcinomatous liver':ti,ab,kw OR 'hepatic cancer':ti,ab,kw OR 'hepatic malignancies':ti,ab,kw OR 'hepatic malignancy':ti,ab,kw OR 'hepatocellular cancer':ti,ab,kw OR 'hepatocellular carcinomatosis':ti,ab,kw OR 'hepatocellular malignancies':ti,ab,kw OR 'hepatocellular malignancy':ti,ab,kw OR 'hepatocyte cancer':ti,ab,kw OR 'liver cancer':ti,ab,kw OR 'liver cell cancer':ti,ab,kw OR 'liver malignancies':ti,ab,kw OR 'liver malignancy':ti,ab,kw OR 'liver primary cancer':ti,ab,kw OR 'malignancies of the liver':ti,ab,kw OR 'malignancy of the liver':ti,ab,kw OR 'malignant hepatic neoplasm':ti,ab,kw OR 'malignant hepatic tumor':ti,ab,kw OR 'malignant hepatic tumour':ti,ab,kw OR 'malignant liver neoplasm':ti,ab,kw OR 'malignant liver tumor':ti,ab,kw OR 'malignant liver tumour':ti,ab,kw OR 'malignant neoplasm of the liver':ti,ab,kw OR 'malignant neoplasms of the liver':ti,ab,kw OR 'malignant tumor of the liver':ti,ab,kw OR 'malignant tumors of the liver':ti,ab,kw OR 'malignant tumour of the liver':ti,ab,kw OR 'malignant tumours of the liver':ti,ab,kw OR 'primary liver cancer':ti,ab,kw OR 'biliary tract cancer'/exp OR 'biliary tract cancer' OR 'biliary cancer':ti,ab,kw OR 'biliary carcinogenesis':ti,ab,kw OR 'biliary malignancies':ti,ab,kw OR 'biliary malignancy':ti,ab,kw OR 'biliary system cancer':ti,ab,kw OR 'biliary tract cancer':ti,ab,kw OR 'cancer of the biliary system':ti,ab,kw OR 'cancer of the biliary tract':ti,ab,kw OR 'malignancies of the biliary tract':ti,ab,kw OR 'malignancy of the biliary tract':ti,ab,kw OR 'malignant tumor of biliary tract':ti,ab,kw OR 'pancreas cancer'/exp OR 'pancreas cancer' OR 'carcinogenesis in the pancreas':ti,ab,kw OR 'carcinogenesis of the pancreas':ti,ab,kw OR 'malignancies of the pancreas':ti,ab,kw OR 'malignancy of the pancreas':ti,ab,kw OR 'malignant neoplasm of the pancreas':ti,ab,kw OR 'malignant pancreatic neoplasm':ti,ab,kw OR 'malignant pancreatic tumor':ti,ab,kw OR 'malignant pancreatic tumour':ti,ab,kw OR 'malignant tumor of the pancreas':ti,ab,kw OR 'malignant tumour of the pancreas':ti,ab,kw OR 'pancreas cancer':ti,ab,kw OR 'pancreas carcinogenesis':ti,ab,kw OR 'pancreas malignancies':ti,ab,kw OR 'pancreas malignancy':ti,ab,kw OR 'pancreatic cancer':ti,ab,kw OR 'pancreatic cancerogenesis':ti,ab,kw OR 'pancreatic carcinogenesis':ti,ab,kw OR 'pancreatic malignancies':ti,ab,kw OR 'pancreatic malignancy':ti,ab,kw OR 'gastrointestinal stromal tumor'/exp OR 'gastrointestinal stromal tumor' OR 'gi stromal tumor':ti,ab,kw OR 'gastric stromal sarcoma':ti,ab,kw OR 'gastro-intestinal stromal tumor':ti,ab,kw OR 'gastro-intestinal stromal tumour':ti,ab,kw OR 'gastrointestinal autonomic nerve tumor':ti,ab,kw OR 'gastrointestinal pacemaker cell tumor':ti,ab,kw OR 'gastrointestinal stroma tumor':ti,ab,kw OR 'gastrointestinal stroma tumour':ti,ab,kw OR 'gastrointestinal stromal neoplasm':ti,ab,kw OR 'gastrointestinal stromal sarcoma':ti,ab,kw OR 'gastrointestinal stromal tumor':ti,ab,kw OR 'gastrointestinal stromal tumors':ti,ab,kw OR 'gastrointestinal stromal tumour':ti,ab,kw OR 'gastrointestinal stromal tumours':ti,ab,kw OR 'liver adenoma'/exp OR 'liver adenoma' OR 'adenoma in the liver':ti,ab,kw OR 'adenoma of the liver':ti,ab,kw OR 'adenoma, liver cell':ti,ab,kw OR 'benign hepatoma':ti,ab,kw OR 'hepatic adenoma':ti,ab,kw OR 'hepatocellular adenoma':ti,ab,kw OR 'liver adenoma':ti,ab,kw OR 'liver cell adenoma':ti,ab,kw) | 1,244,538 |
| #3 | #1 AND #2 | 7,249 |
| **SCOPUS** | | |
| #1 | (TITLE-ABS-KEY("gamma-Glutamyltransferase" OR “gamma Glutamyltransferase” OR “GGTP” OR “gammaglutamyltransferase” OR “gamma-Glutamyl Transpeptidase” OR “Transpeptidase, gamma-Glutamyl” OR “Transpeptidase, gamma-Glutamyl” OR “gamma Glutamyl Transpeptidase” OR “Glutamyl Transpeptidase” OR “Transpeptidase, Glutamyl”)) | 62,699 |
| #2 | (TITLE-ABS-KEY(“mouth cancer” OR “mouth cancer” OR “cancer, mouth” OR “intraoral cancer” OR “mouth cancer” OR “mouth mucosa cancer” OR “oral cancer” OR “oral cavity cancer” OR “oropharynx cancer” OR “oropharynx cancer” OR “cancer, oropharyngeal” OR “oropharyngeal cancer” OR “oropharynx cancer” OR “esophagus cancer” OR “esophagus cancer” OR “cancer of the abdominal esophagus” OR “cancer of the cervical esophagus” OR “cancer of the cervical oesophagus” OR “cancer of the esophagus” OR “cancer of the oesophagus” OR “cancer of the thoracic esophagus” OR “cancer of the thoracic oesophagus” OR “cancer, esophagus” OR “carcinogenesis in the esophagus” OR “esophageal cancer” OR “esophageal carcinogenesis” OR “esophageal malignancies” OR “esophageal malignancy” OR “esophagus cancer” OR “malignancies of the esophagus” OR “malignancies of the oesophagus” OR “malignancy of the esophagus” OR “malignancy of the oesophagus” OR “malignant esophageal disease” OR “malignant esophageal tumor” OR “malignant neoplasm of the esophagus” OR “malignant neoplasm of the oesophagus” OR “malignant oesophageal disease” OR “malignant tumor of the esophagus” OR “oesophageal cancer” OR “oesophageal carcinogenesis” OR “oesophageal malignancies” OR “oesophageal malignancy” OR “oesophagus cancer” OR “stomach cancer” OR “stomach cancer” OR “cancer of the cardia” OR “cancer of the gastric antrum” OR “cancer of the gastric body” OR “cancer of the gastric cardia” OR “cancer of the gastric fundus” OR “cancer, stomach” OR “cardia cancer” OR “gastric antral cancer” OR “gastric antrum cancer” OR “gastric body cancer” OR “gastric cancer” OR “gastric cardia cancer” OR “gastric cardiac cancer” OR “gastric malignancies” OR “gastric malignancy” OR “malignancies of the stomach” OR “malignancy of the stomach” OR “malignant gastric neoplasm” OR “malignant gastric tumor” OR “malignant neoplasm of the stomach” OR “malignant neoplasms of the stomach” OR “malignant tumor of the stomach” OR “malignant tumors of the stomach” OR “malignant tumour of the stomach” OR “malignant tumours of the stomach” OR “pyloric cancer” OR “stomach cancer” OR “stomach malignancies” OR “stomach malignancy” OR “intestine cancer” OR “intestine cancer” OR “bowel cancer” OR “cancer of the bowel” OR “cancer of the intestine” OR “cancer, intestine” OR “gut cancer” OR “gut carcinogenesis” OR “gut malignancy” OR “intestinal cancer” OR “intestinal cancerogenesis” OR “intestinal carcinogenesis” OR “intestinal malignancies” OR “intestinal malignancy” OR “intestine cancer” OR “intestine carcinogenesis” OR “intestine malignancies” OR “intestine malignancy” OR “malignant intestinal neoplasm” OR “duodenum cancer” OR “duodenum cancer” OR “cancer of the duodenal” OR “cancer of the duodenum” OR “duodenal cancer” OR “duodenal carcinogenesis” OR “duodenal malignancies” OR “duodenal malignancy” OR “duodenum cancer” OR “jejunum cancer” OR “jejunum cancer” OR “cancer of the jejunum” OR “cancer, jejunum” OR “jejunal cancer” OR “jejunal carcinogenesis” OR “jejunal malignancy” OR “jejunum cancer” OR “malignant tumor of the jejunum” OR “ileum cancer” OR “ileum cancer” OR “cancer of the ileum” OR “cancer, ileum” OR “ileal cancer” OR “ileum cancer” OR “malignant ileal” OR “cecum cancer” OR “cecum cancer” OR “caecal cancer” OR “caecum cancer” OR “cancer of the cecum” OR “cancer, cecum” OR “cecal cancer” OR “cecum cancer” OR “cecum sarcoma” OR “colorectal cancer” OR “colorectal cancer” OR “cancer of colon and rectum” OR “cancer of rectum and colon” OR “cancer of the colon and rectum” OR “cancer of the colon and the rectum” OR “cancer of the rectum and colon” OR “cancer of the rectum and the colon” OR “colo-rectal cancer” OR “colo-rectal carcinogenesis” OR “colo-rectal malignancies” OR “colo-rectal malignancy” OR “colorectal cancer” OR “colorectal cancerogenesis” OR “colorectal carcinogenesis” OR “colorectal malignancies” OR “colorectal malignancy” OR “malignancies of the colon and rectum” OR “malignancy of colon and rectum” OR “malignancy of the colon and rectum” OR “recto-colonic cancer” OR “rectocolonic cancer” OR “liver cancer” OR “liver cancer” OR “cancer of the liver” OR “cancer, liver” OR “carcinomatous liver” OR “hepatic cancer” OR “hepatic malignancies” OR “hepatic malignancy” OR “hepatocellular cancer” OR “hepatocellular carcinomatosis” OR “hepatocellular malignancies” OR “hepatocellular malignancy” OR “hepatocyte cancer” OR “liver cancer” OR “liver cell cancer” OR “liver malignancies” OR “liver malignancy” OR “liver primary cancer” OR “malignancies of the liver” OR “malignancy of the liver” OR “malignant hepatic neoplasm” OR “malignant hepatic tumor” OR “malignant hepatic tumour” OR “malignant liver neoplasm” OR “malignant liver tumor” OR “malignant liver tumour” OR “malignant neoplasm of the liver” OR “malignant neoplasms of the liver” OR “malignant tumor of the liver” OR “malignant tumors of the liver” OR “malignant tumour of the liver” OR “malignant tumours of the liver” OR “primary liver cancer” OR “biliary tract cancer” OR “biliary tract cancer” OR “biliary cancer” OR “biliary carcinogenesis” OR “biliary malignancies” OR “biliary malignancy” OR “biliary system cancer” OR “biliary tract cancer” OR “cancer of the biliary system” OR “cancer of the biliary tract” OR “malignancies of the biliary tract” OR “malignancy of the biliary tract” OR “malignant tumor of biliary tract” OR “pancreas cancer” OR “pancreas cancer” OR “carcinogenesis in the pancreas” OR “carcinogenesis of the pancreas” OR “malignancies of the pancreas” OR “malignancy of the pancreas” OR “malignant neoplasm of the pancreas” OR “malignant pancreatic neoplasm” OR “malignant pancreatic tumor” OR “malignant pancreatic tumour” OR “malignant tumor of the pancreas” OR “malignant tumour of the pancreas” OR “pancreas cancer” OR “pancreas carcinogenesis” OR “pancreas malignancies” OR “pancreas malignancy” OR “pancreatic cancer” OR “pancreatic cancerogenesis” OR “pancreatic carcinogenesis” OR “pancreatic malignancies” OR “pancreatic malignancy” OR “gastrointestinal stromal tumor” OR “gastrointestinal stromal tumor” OR “gi stromal tumor” OR “gastric stromal sarcoma” OR “gastro-intestinal stromal tumor” OR “gastro-intestinal stromal tumour” OR “gastrointestinal autonomic nerve tumor” OR “gastrointestinal pacemaker cell tumor” OR “gastrointestinal stroma tumor” OR “gastrointestinal stroma tumour” OR “gastrointestinal stromal neoplasm” OR “gastrointestinal stromal sarcoma” OR “gastrointestinal stromal tumor” OR “gastrointestinal stromal tumors” OR “gastrointestinal stromal tumour” OR “gastrointestinal stromal tumours” OR “liver adenoma” OR “liver adenoma” OR “adenoma in the liver” OR “adenoma of the liver” OR “adenoma, liver cell” OR “benign hepatoma” OR “hepatic adenoma” OR “hepatocellular adenoma” OR “liver adenoma” OR “liver cell adenoma”)) | 609,952 |
| #3 | #1 AND #2 | 2,392 |
| **Total records** | | **12,182** |
| **Total records without duplicates** | | **8,747** |

***
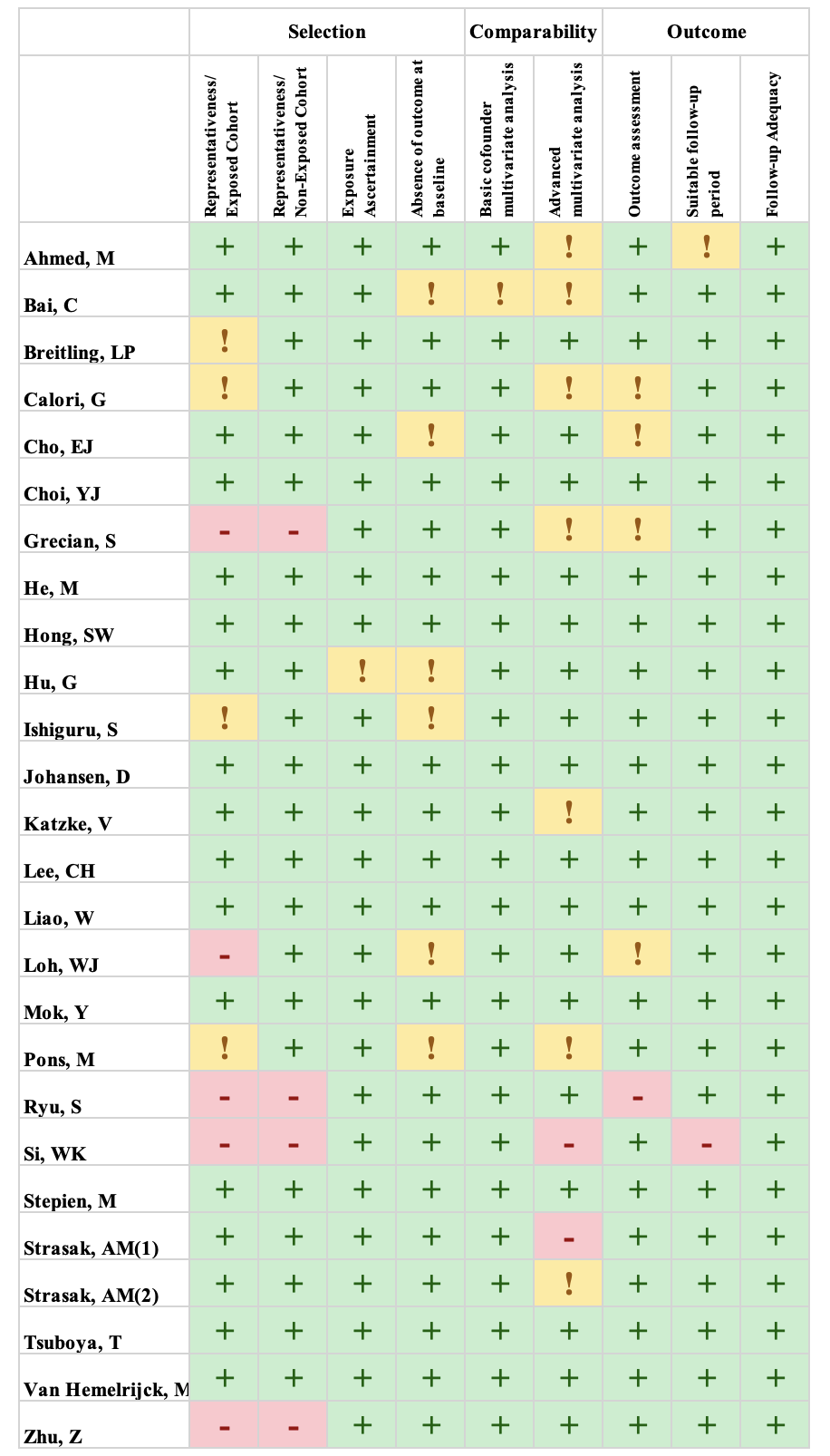

Supplementary Figure 1****. Quality assessment based on New-Castle Ottawa scale*

***
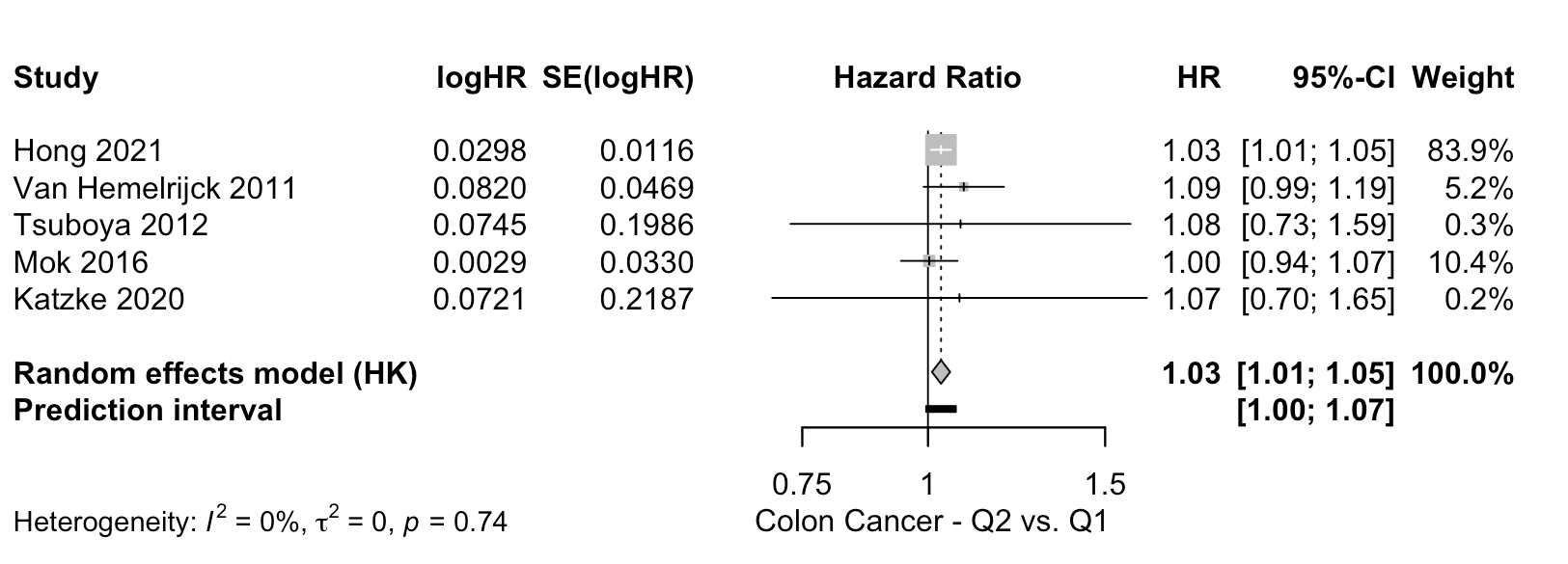
***

***Supplementary Figure 2.*** *Forest plot for meta-analysis of colorectal cancer risk in Q2 of GGT, compared to Q1*

*
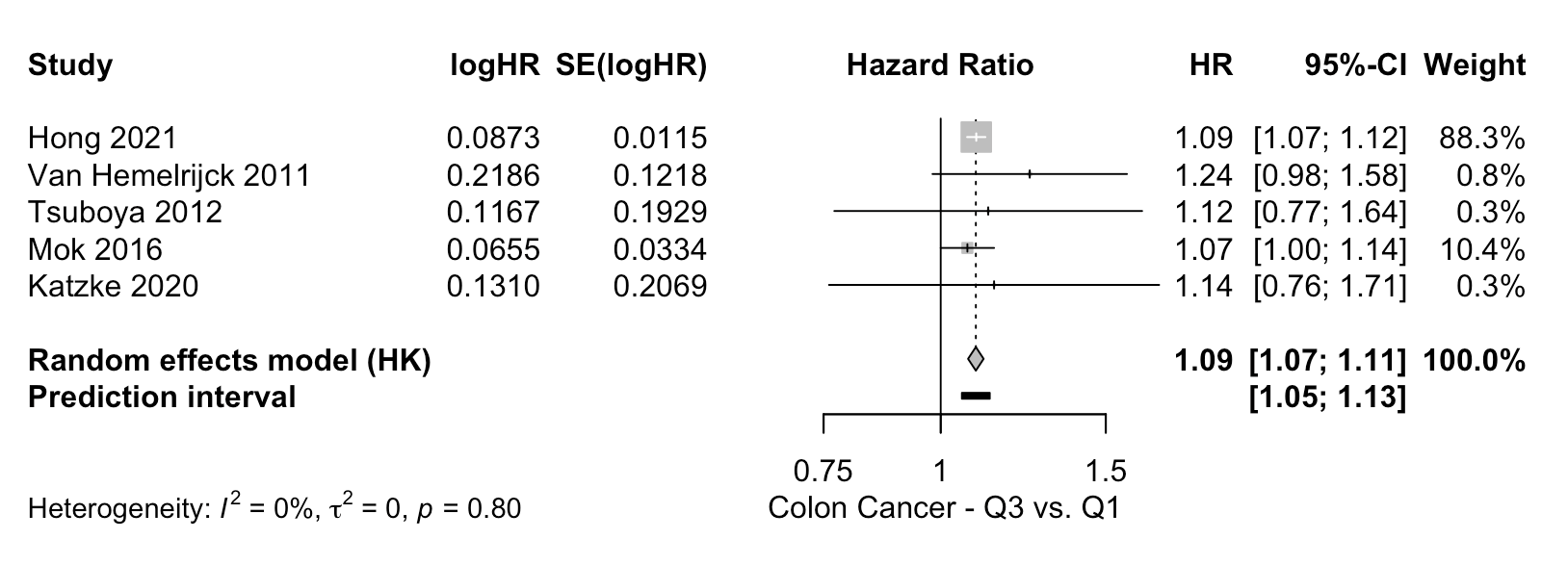
*

***Supplementary Figure 3.*** *Forest plot for meta-analysis of colorectal cancer risk in Q3 of GGT, compared to Q1*

*
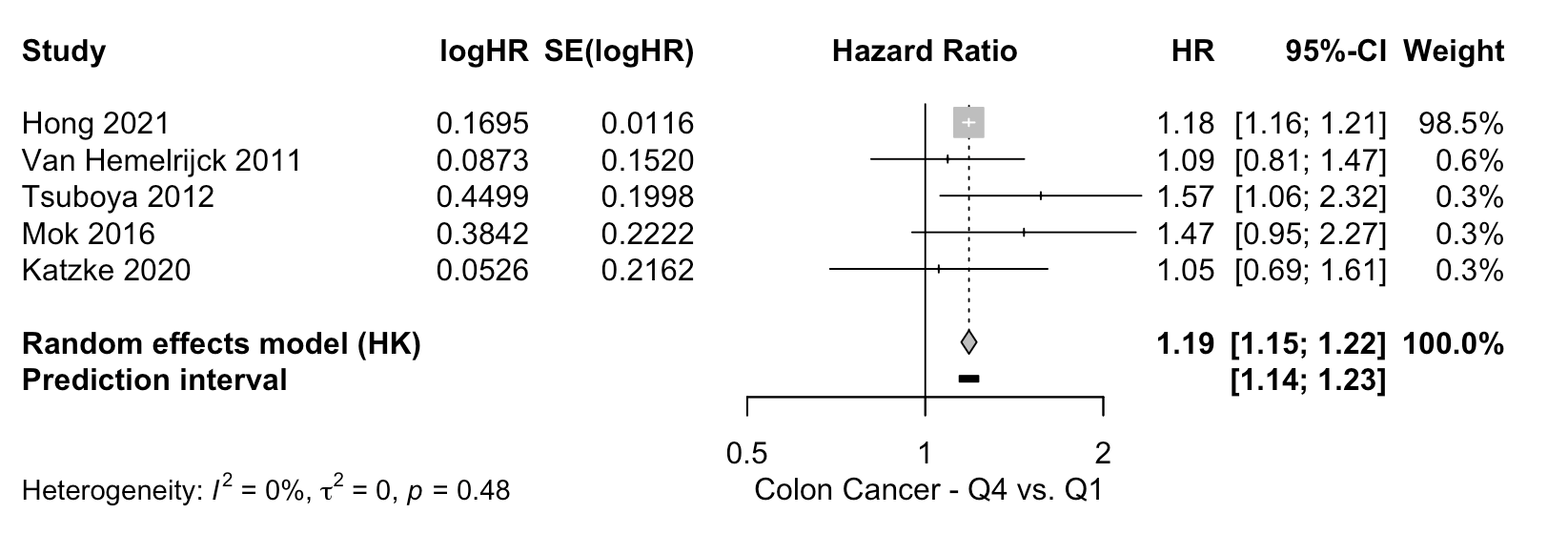
*

***Supplementary Figure 4.*** *Forest plot for meta-analysis of colorectal cancer risk in Q4 of GGT, compared to Q1*

*
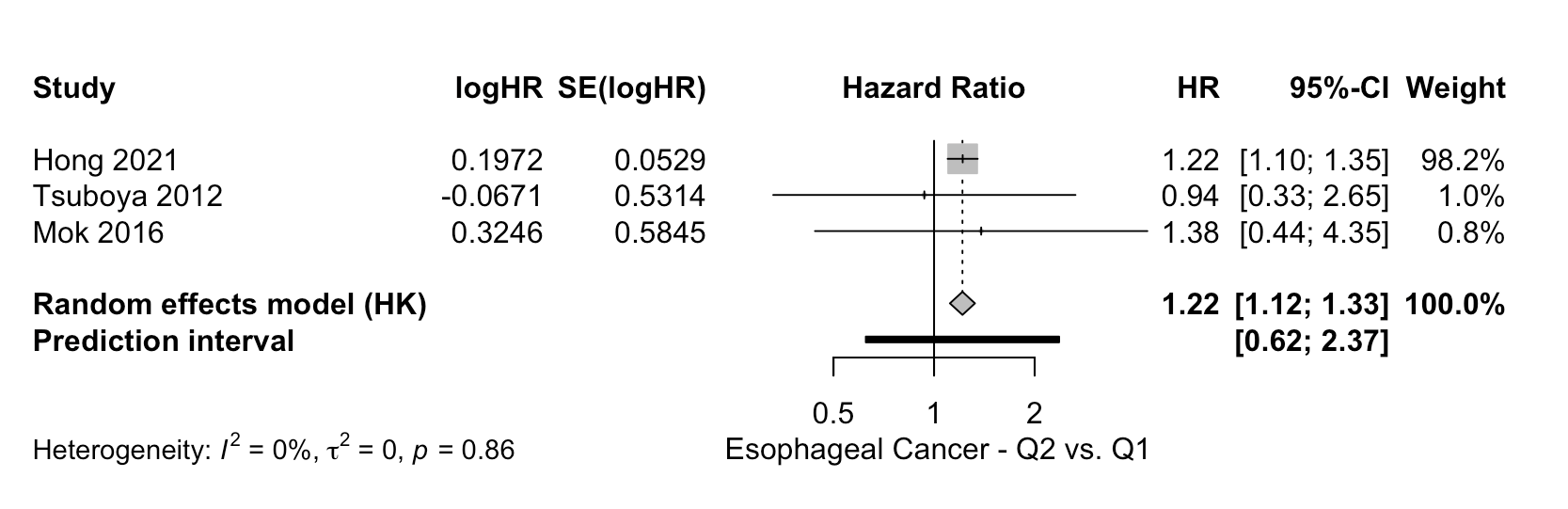
*

***Supplementary Figure 5.*** *Forest plot for meta-analysis of esophageal cancer risk in Q2 of GGT, compared to Q1*

*
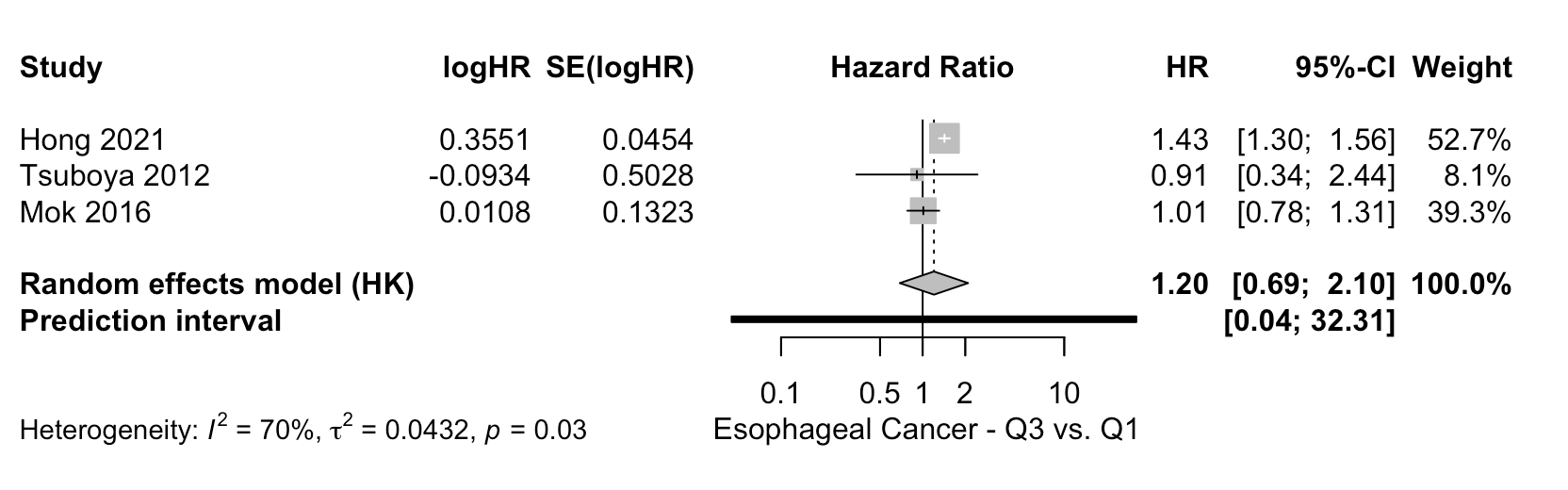
*

***Supplementary Figure 6.*** *Forest plot for meta-analysis of esophageal cancer risk in Q3 of GGT, compared to Q1*

*
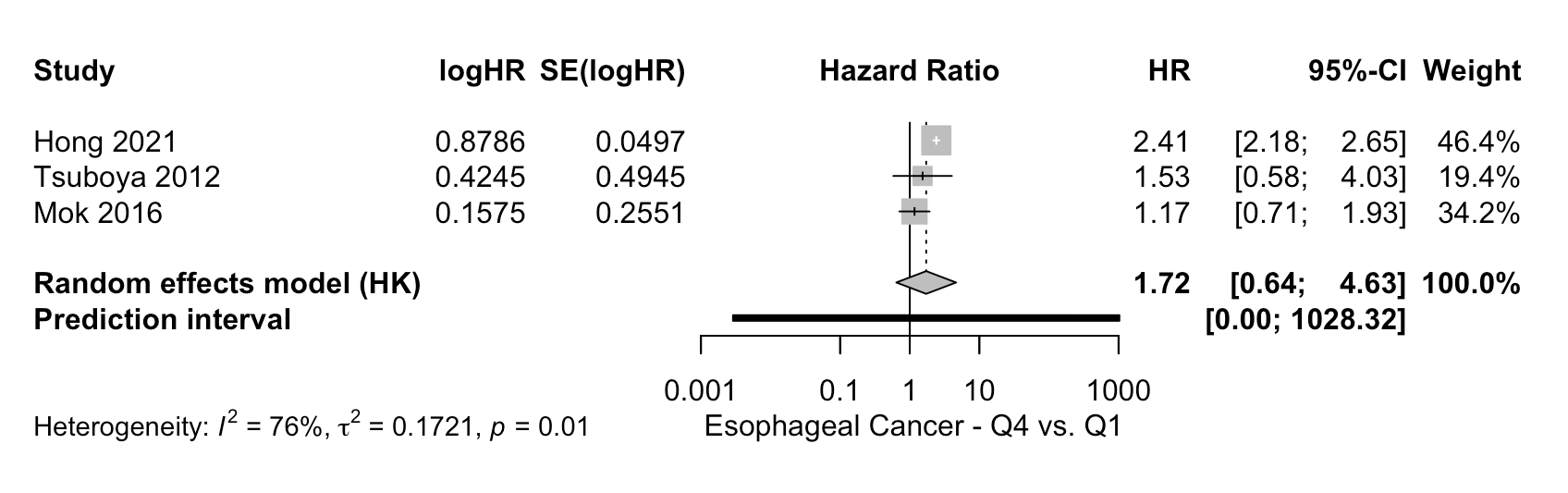
*

***Supplementary Figure7.*** *Forest plot for meta-analysis of esophageal cancer risk in Q4 of GGT, compared to Q1*

***
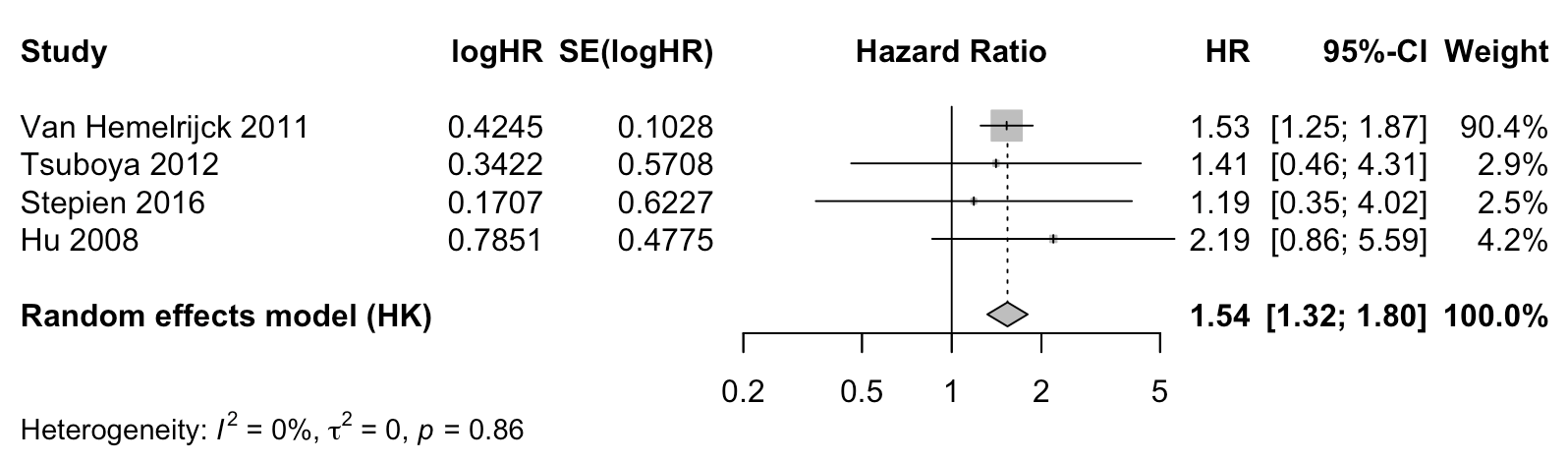
Supplementary Figure 8.*** *Forest plot for meta-analysis of liver cancer risk in Q2 of GGT, compared to Q1*

*
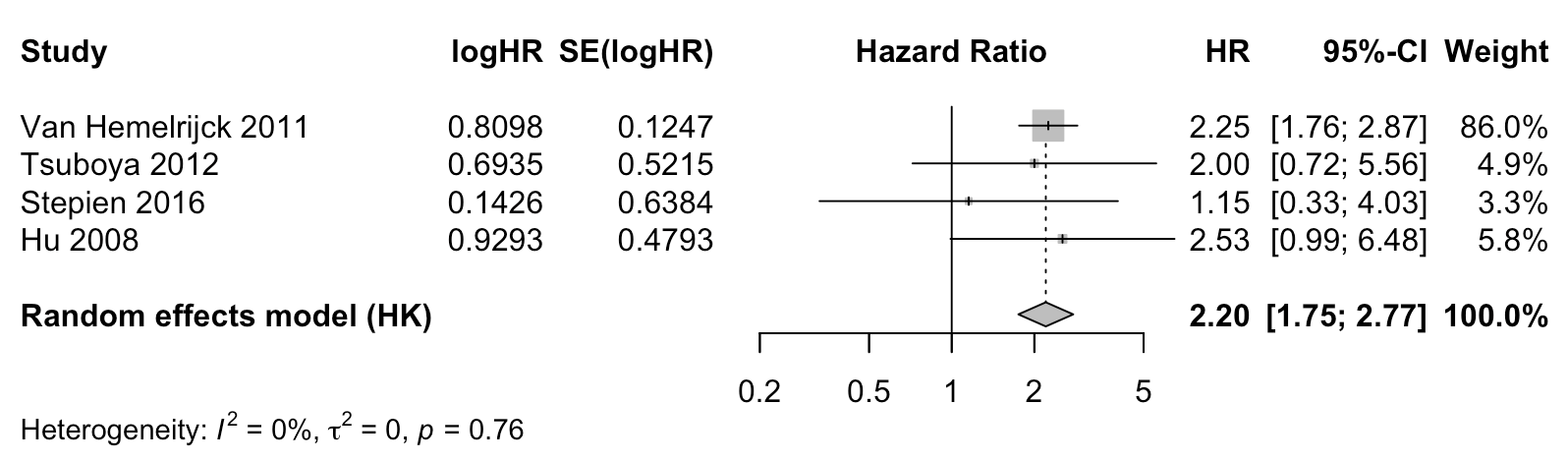
*

***Supplementary Figure 9.*** *Forest plot for meta-analysis of liver cancer risk in Q3 of GGT, compared to Q1*

*
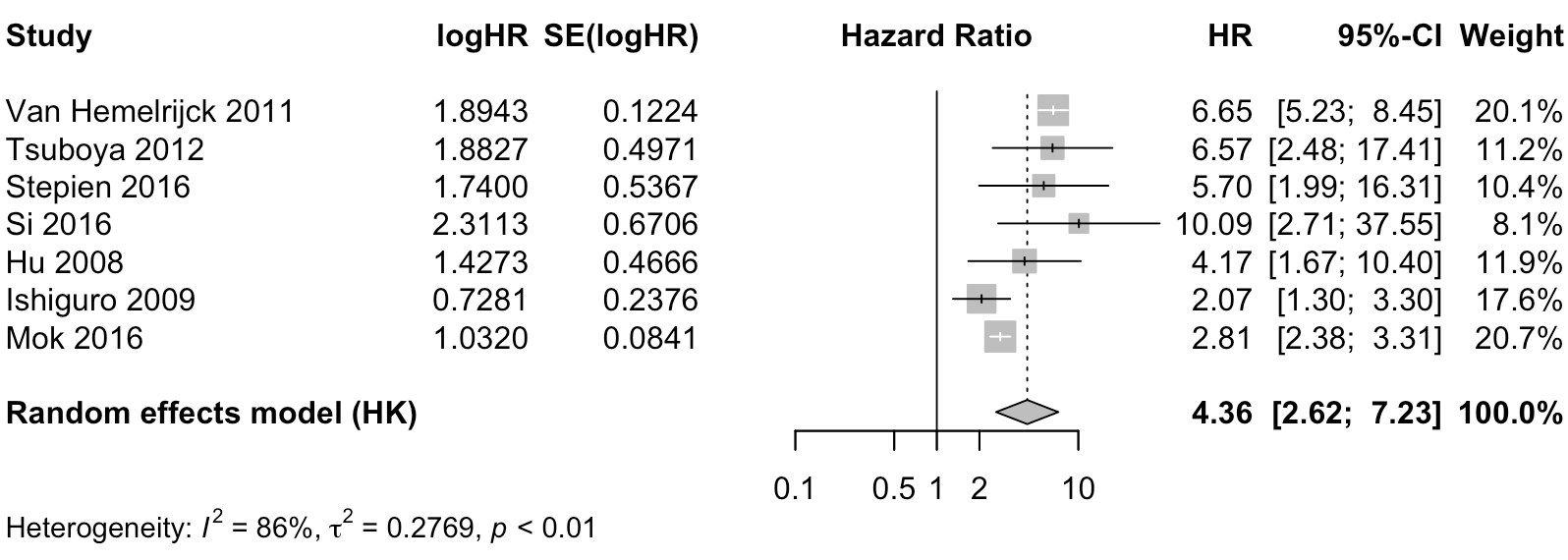
*

***Supplementary Figure 10.*** *Forest plot for meta-analysis of liver cancer risk in Q4 of GGT, compared to Q1*

*
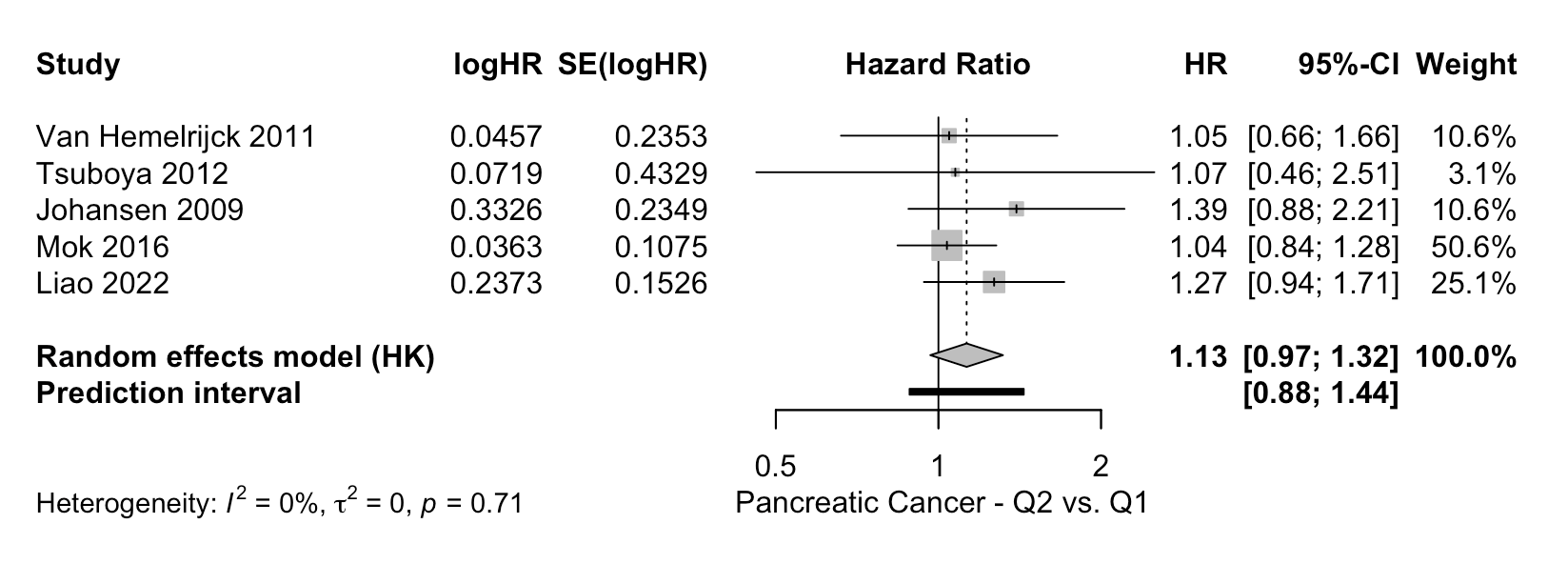
*

***Supplementary Figure 11.*** *Forest plot for meta-analysis of pancreas cancer risk in Q2 of GGT, compared to Q1*

*
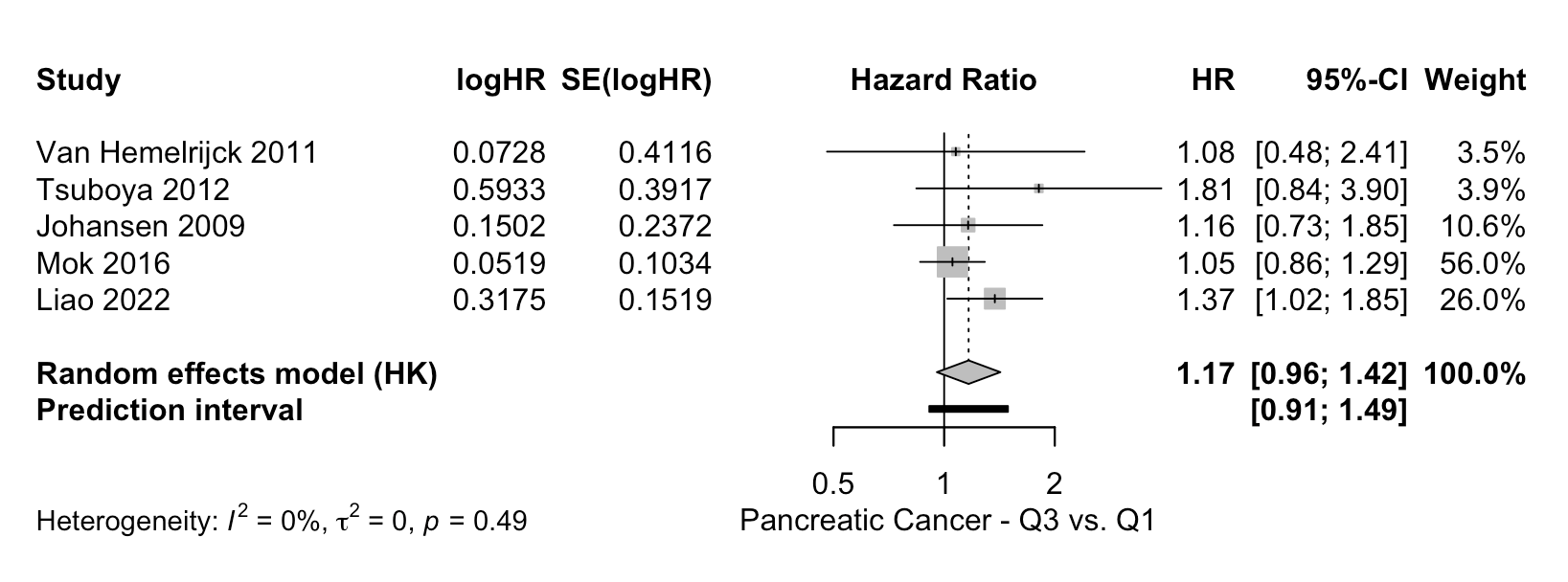
*

***Supplementary Figure 12.*** *Forest plot for meta-analysis of pancreas cancer risk in Q3 of GGT, compared to Q1*

*
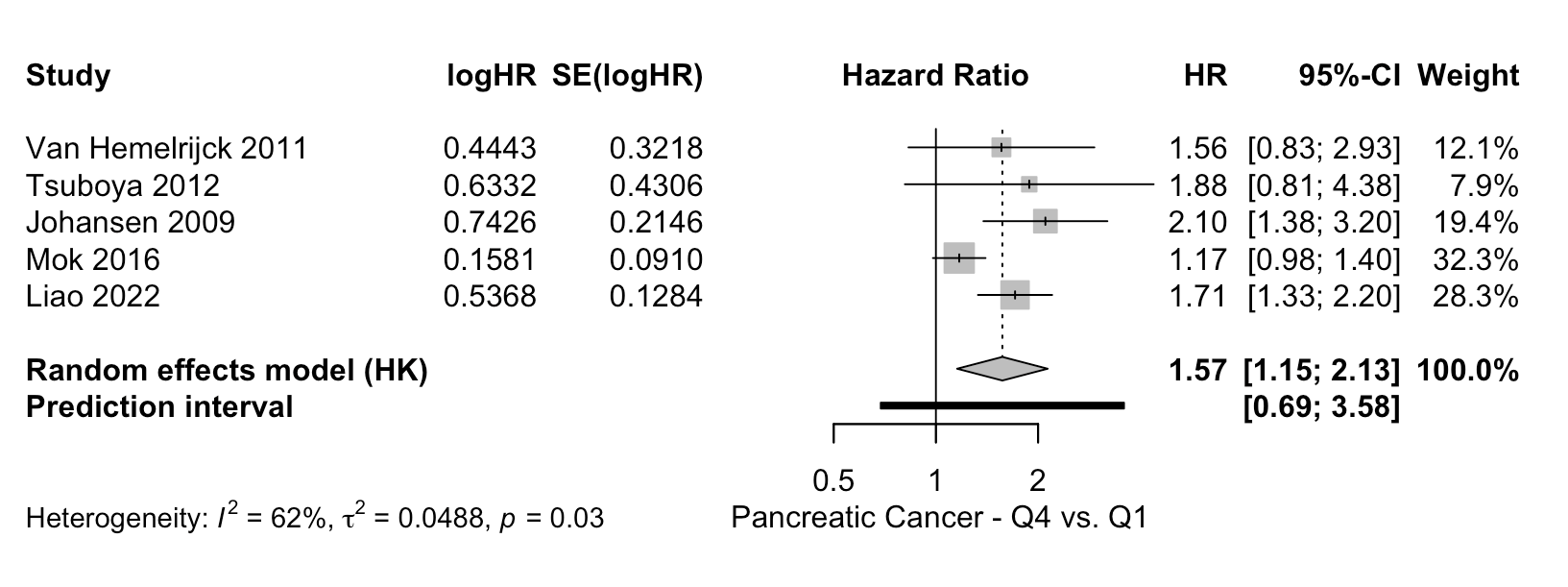
*

***Supplementary Figure 13.*** *Forest plot for meta-analysis of pancreas cancer risk in Q4 of GGT, compared to Q1*

*
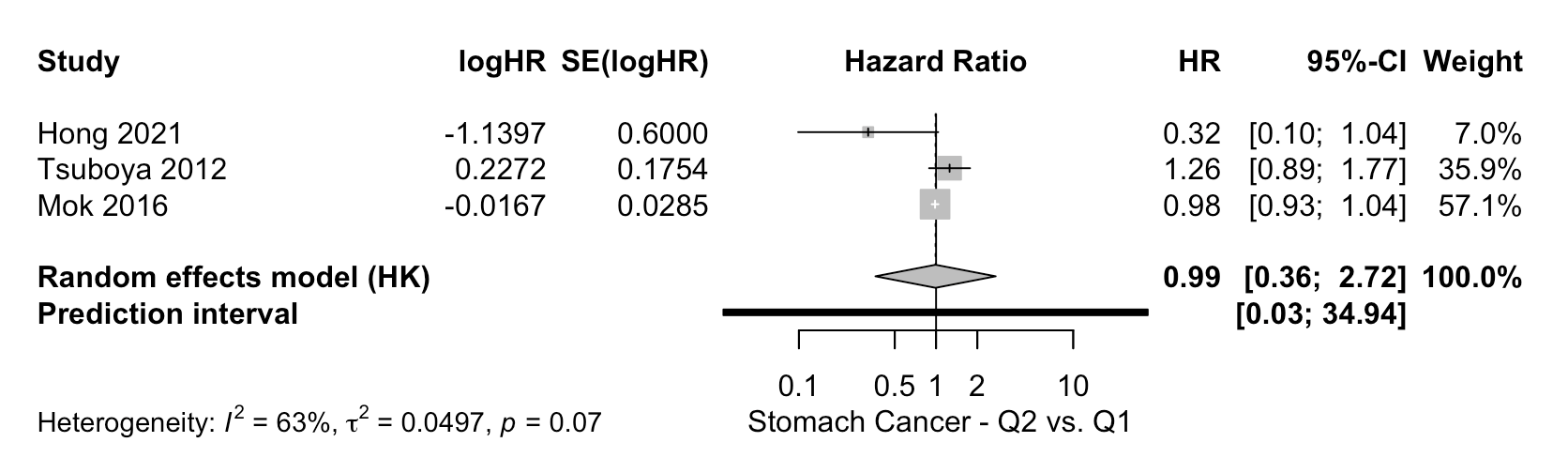
*

***Supplementary Figure 14.*** *Forest plot for meta-analysis of gastric cancer risk in Q2 of GGT, compared to Q1*

*
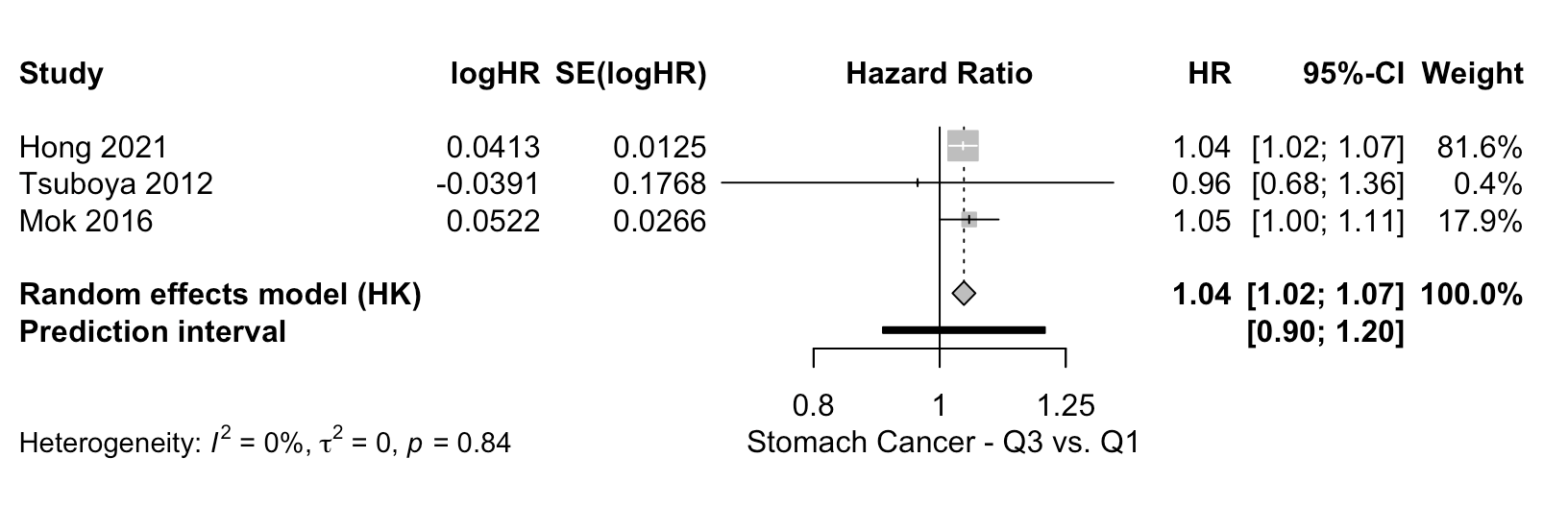
*

***Supplementary Figure 15.*** *Forest plot for meta-analysis of gastric cancer risk in Q3 of GGT, compared to Q1*

*
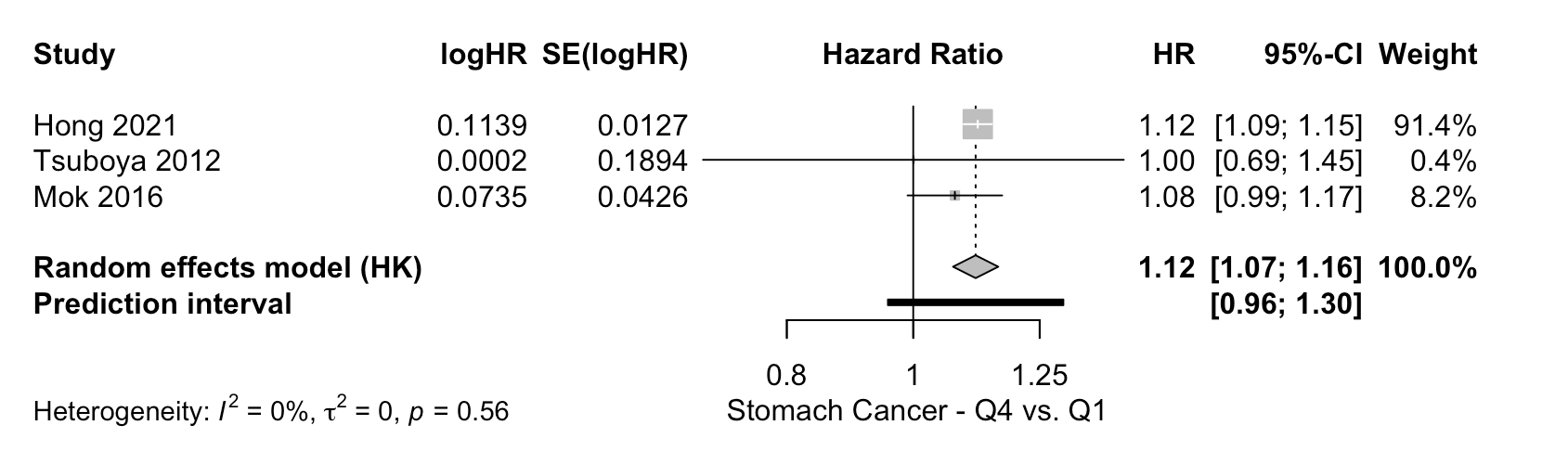
*

***Supplementary Figure 16.*** *Forest plot for meta-analysis of gastric cancer risk in Q4 of GGT, compared to Q1*
